# A new mechanism regulating microglial NLRP3 inflammasome: FMR1 mediates NLRP3 mRNA stability

**DOI:** 10.1371/journal.pone.0341867

**Published:** 2026-02-20

**Authors:** Qian Deng, Qianqian Bai, Yang Yang, Wenchao Tang, Fangfei Liu, Shumin Zhang, Hao Wang, Zhehua Xing, Chi Zhang, Yanhui Yang, Qizhi Fu, Hua Fan

**Affiliations:** 1 Department of Pharmacy, Luoyang Central Hospital Affiliated to Zhengzhou University, Luoyang, China; 2 The First Affiliated Hospital, and College of Clinical Medicine of Henan University of Science and Technology, Luoyang, China; 3 Department of Trauma center, The First Affiliated Hospital, College of Clinical Medicine, Henan University of Science and Technology, Luoyang, China; 4 Department of Intensive Care Unit (Internal Medicine), The First Affiliated Hospital, And College of Clinical Medicine of Henan University of Science and Technology, Luoyang, China; 5 Luoyang Key Laboratory of Neuroimmunology and innovative Drug Screening, Luoyang, China; Jiaxing University, CHINA

## Abstract

**Background:**

The NLRP3 inflammasome drives chronic inflammation and contributes to the pathogenesis of multiple sclerosis (MS). This study aimed to elucidate a novel post-transcriptional regulatory mechanism controlling NLRP3 expression in microglia under lipopolysaccharide (LPS) stimulation.

**Methods:**

The experimental autoimmune encephalomyelitis (EAE) mouse model of MS was established and divided into four groups: Sham, EAE, EAE + Lv-con (control lentivirus), and EAE + Lv-FMR1 (FMR1-overexpressing lentivirus). BV2 microglial cells were stimulated with LPS and adenosine triphosphate (ATP). mRNA and protein levels were assessed by qPCR, western blot, immunofluorescence, and immunohistochemistry. Caspase-1 activity and IL-1β/IL-18 levels were quantified using commercial assay kits. RNA-binding proteins (RBPs) interacting with NLRP3 mRNA were identified by RNA pull-down combined with mass spectrometry. NLRP3 mRNA stability was analyzed using Actinomycin D.

**Results:**

NLRP3 inflammasome activation was confirmed in the spinal cords of EAE mice and in LPS/ATP-stimulated BV2 cells. Lentivirus-mediated overexpression of FMR1 in EAE mice attenuated microglial activation (reduced IBA-1) and decreased NLRP3 expression compared to the EAE + Lv-con control group. Immunohistochemistry confirmed reduced caspase-1 deposition in the EAE + Lv-FMR1 group. Mechanistically, FMR1 directly interacted with the 3’ untranslated region (3’UTR) of NLRP3 mRNA in LPS/ATP-treated BV2 cells, leading to mRNA destabilization and consequent suppression of NLRP3 protein expression. Functionally, FMR1 inhibited NLRP3 inflammasome activation by downregulating NLRP3.

**Conclusion:**

FMR1 suppresses NLRP3 inflammasome activation in both EAE mice and microglial cell models by destabilizing NLRP3 mRNA. This suggests that FMR1 possesses therapeutic potential for MS by dually regulating neuroinflammation and NLRP3-driven pathology.

## Introduction

Multiple sclerosis (MS) is a chronic demyelinating disease that affects approximately 2 million people worldwide. It is characterized by immune-mediated damage to the myelin sheaths in the central nervous system (CNS), leading to neurodegeneration and cognitive decline [1]. Microglia, the resident immune cells of the CNS, play dual roles in neuroinflammation and repair. However, their dysregulation significantly exacerbates MS pathology [2]. Despite advances in therapy, current treatments remain inadequate in preventing neurodegeneration.

The NLRP3 inflammasome, a multiprotein complex comprising NLRP3, ASC, and caspase-1, drives the maturation of pro-inflammatory cytokines IL-1β and IL-18 and executes pyroptosis, an inflammatory form of programmed cell death [3]. Aberrant activation of the NLRP3 inflammasome can induce a chronic inflammatory state and has been implicated in the pathogenesis of various neurological disorders, including MS [4]. Elevated expression of NLRP3 is observed in both MS patients and the experimental autoimmune encephalomyelitis (EAE) mouse model [5,6]. Consistently, *Nlrp3*^*-/-*^ mice exhibit reduced inflammatory cell infiltration and markedly mild EAE symptoms [7]. Furthermore, elevated levels of caspase-1 and IL-1β are detected in MS mouse models [8]. Microglial NLRP3 activation correlates with the severity of demyelination, and IL-1β amplifies neuroinflammation in MS [5]. In addition to IL-1β, IL-18 is also highly expressed in LPS- and ATP-challenged microglia [9]. Moreover, in the cuprizone-induced model of MS, nebivolol exerts neuroprotective effects by suppressing the NLRP3/caspase-1/IL-18 cascade [10]. These findings collectively underscore the critical involvement of NLRP3 inflammasome components and their downstream cytokines, IL-1β and IL-18, in MS.

In recent years, the development of effective NLRP3 inflammasome inhibitors for MS treatment has been an active area of investigation [5,11]. Some research efforts have focused on identifying key upstream modulators that directly regulate NLRP3 via post-transcriptional mechanisms. Among these modulators, RNA binding proteins (RBPs) have attracted considerable attention [12–14]. Fragile X Mental Retardation 1 (FMR1), an RBP linked to neurodevelopmental disorders, is also known to modulate inflammatory responses [15]. During *Drosophila* embryogenesis, FMR1 facilitates the degradation of maternal RNAs by preferentially binding to target mRNAs containing an m6A-modified “AGACU” motif [16]. The involvement of FMR1 in neurodevelopmental diseases is well-established, and the *Fmr1*^-/-^ microglia display enhanced pro-inflammatory responses following lipopolysaccharide (LPS) exposure [17]. Notably, FMR1 premutation has been reported in MS patients [18], and bioinformatics analyses implicate FMR1 in MS pathogenesis [19], suggesting a potential role in neuroinflammation.

To robustly activate the NLRP3 inflammasome *in vitro*, a well-established two-signal model is commonly employed. The first signal (LPS, a TLR4 agonist) acts as a priming stimulus that upregulates the transcription of NLRP3 and pro-IL-1β via the NF-κB pathway. The second signal (ATP, a P2X7 receptor agonist) provides the activation stimulus that triggers NLRP3 oligomerization and inflammasome assembly. LPS has been confirmed to activate the NLRP3 inflammasome under ATP in various cellular models [20–22]. Based on this model, the present study aimed to unveil a novel post-transcriptional mechanism controlling NLRP3 and its inflammasome in LPS/ATP-challenged BV2 microglia. We hypothesized that FMR1 acts as a key regulator in this process. Elucidating this molecular basis may identify promising NLRP3 inflammasome inhibitors for the prevention and treatment of MS.

## Materials and methods

### Mice and EAE induction

Female wild-type C57BL/6 mice (8 weeks old, 18–22 g) were obtained from Jiangsu Huachuang Sinno Pharmaceutical Technology and maintained under specific pathogen-free (SPF) conditions with 12-h light/dark cycles, 22 ± 1°C, 55% humidity, and ad libitum access to food/water. Female predisposition in this strain parallels higher MS incidence in women, enhancing translational relevance. No unanticipated complications occurred. All procedures followed National guidelines for Use and Care of animals and were approved by the Institutional Animal Care and Use Committee (IACUC) of the First Affiliated Hospital of Henan University of Science and Technology (Approval No. D-2023–026). This study followed ARRIVE 2.0 guidelines but was not preregistered in a public repository. Six mice per group (total n = 12 for EAE and Sham groups) were randomly assigned to EAE or Sham groups using a random number table. Sample size was determined based on previous EAE studies, without formal power calculation. Inclusion criteria required baseline weight ≥ 18g and normal neurological function (score = 0). Animals failing to develop EAE (score <2 by day 14) would be excluded, though all met inclusion criteria. No animals or data points were excluded from analysis, and three mice per group were randomly selected for histological assays. The EAE model in C57BL/6 mice recapitulates key MS features including distinct inflammatory infiltration in the spinal cord. For EAE model generation, mice were used under standard protocols as described [23]. Briefly, an emulsion for *in vivo* immunization was first made, which included myelin oligodendrocyte glycoprotein 35–55 (MOG_35–55_), (#4010006243, China Peptides, Shanghai, China) emulsified (1:1) in complete Freund’s adjuvant (CFA), (#F5881, Sigma-Aldrich, St. Louis, MO, USA) containing inactivated H37RA strain mycobacterium tuberculosis (#231141, BD Diagnostics, Frankilin Lakes, NJ, USA). The EAE model was induced by subcutaneous immunization with the suitable volume of the emulsion to ensure 200 µg MOG_35–55_ for each mouse, followed by the intraperitoneal injection of pertussis toxin (0.2 µg per mouse, #P2980, Sigma-Aldrich, St. Louis, MO, USA) at days 0 and 2 after subcutaneous immunization. During immunization, mice were anesthetized with 3% isoflurane to minimize pain. The mice in the Sham group were given the same volume of PBS. Neurological scoring was performed using the following scale: 0, no disease; 1, limp tail; 2, hindlimb weakness; 3, hindlimb paralysis; 4, forelimb weakness or paralysis; 5, moribund or death. The peak clinical score for EAE group was 3.82 ± 0.41, and 0.00 ± 0.00 for Sham group. All procedures (immunizations, scoring) were performed in randomized order between 9:00–11:00 AM by the same operator. Pain management included the use of 3% isoflurane anesthesia during the intraoperative phase. Postoperatively, buprenorphine was administered subcutaneously at a dose of 0.1 mg/kg every 12 h for 48 h. Additionally, extended analgesia was provided for mice with pain scores of 3 or higher. The study spanned 24 days post-immunization (day 0 to day 24). Mice were monitored daily for clinical scores (0–5) based on the grading standards as described [23], weight, and abnormalities. Those with scores≥3 received twice-daily assessments and soft food/hydration gels. The method of sacrifice was intraperitoneal injection of sodium pentobarbital (150 mg/kg). This method ensured death by respiratory and cardiac arrest, which was verified by the absence of a heartbeat and pupillary reflex. All efforts were made to minimize suffering. This method was applied to animals that reached humane endpoints (weight loss >20% or a clinical score of 5) within 2 h, as well as to all remaining survivors at the end of the study (day 24 post-immunization). No animals died before meeting predefined euthanasia criteria. Neurological scoring was designated as the primary endpoint for sample size determination, as it directly quantifies EAE disease severity [23]. The limitation of animal study includes the set of endpoint, and endpoint analysis at day 24 might miss early inflammasome priming events. No blinding was conducted in this study. All personnel completed training programs in rodent handling, anesthesia administration, and euthanasia techniques. Spinal cord samples were utilized for histology, immunofluorescence and immunohistochemical staining analyses.

### Histology, immunofluorescence and immunohistochemical staining

Sections at 4 µm thickness of paraffin-embedded spinal cord were subjected to hematoxylin and eosin (H&E) staining under standard methods. Immunofluorescence of ionised calcium binding adaptor molecule 1 (IBA-1) and NLRP3 and immunohistochemical staining of caspase-1 were conducted on paraffin-embedded spinal cord sections, which were deparaffinized and subjected to antigen retrieval (boiling; 15 min) in sodium citrate (pH 6.0), as well as blocking (room temperature; 30 min) in 3% BSA (#SW3015, Solarbio, Beijing, China). Expression of IBA-1 or NLRP3 in mouse spinal cord was probed with anti-IBA-1 rabbit pAb (#GB11105, 1:800, Servicebio, Wuhan, China) or anti-NLRP3 rabbit pAb (#GB114320, 1:300, Servicebio). Expression of caspase-1 in mouse spinal cord was probed with anti-caspase-1 rabbit pAb (#22915–1-AP, 1:250, Proteintech, Wuhan, China). Incubation with anti-rabbit secondary antibody labeled by Cy3 (for immunofluorescence; #GB21303, 1:500, Servicebio) or HRP (for immunohistochemistry; #ab6721, 1:1000, Abcam, Cambridge, UK) was then carried out. In immunofluorescence assay, DAPI was used for nuclear staining. For immunohistochemical staining, the DAB HRP Color Development Kit (#P0202) was applied as described by the vendor (Beyotime, Shanghai, China). Images were captured and analyzed for IBA-1 or NLRP3 fluorescence intensity or the depicted area of caspase-1 using ImageJ (NIH, Bethesda, MD, USA).

### Cell culture and treatment

Mouse BV2 microglia (#ml96448, Mlbio, Shanghai, China) were grown in the special medium (#CM-0493A, Procell, Wuhan, China), which included DMEM, 1% streptomycin/penicillin, and 10% FBS, in the water-saturated 5% CO_2_ incubator at 37°C. LPS (98% purity, #S7850, Selleck, Shanghai, China) diluted in PBS vehicle was added into the culture media at the 100 ng/mL final concentration and applied for 24 h, followed by treatment of ATP (5 mM, #A3377, Sigma-Aldrich) for 30 min. PBS vehicle alone was applied as the control. MCC950 (99.7% purity, #S8930, Selleck), a specific suppressor of the NLRP3 [24], diluted in DMSO vehicle solution or vehicle mock was pre-added to the media and applied for 2 h before LPS/ATP addition and treatment.

### Generation of FMR1 and NLRP3 overexpressing BV2 microglia

To overexpress FMR1, the pEnCMV-Fmr1(mouse)-V5-IRES2-EGFP-Puro (#P29807, Miaoling, Wuhan, China) was employed. To upregulate NLRP3, the pCMV-Nlrp3(mouse)-3 × Myc-Neo (#P55143, Miaoling) was applied. As control, the negative pCMV vector was also obtained from Miaoling. Overexpressing BV2 microglia cells were generated as follows: 3.0 × 10^4^ BV2 cells were plated into a 24-multiwell white plastic plate 18–24 h before transaction. The second day, 0.2 µg FMR1 expression plasmid alone, 0.1 µg FMR1 plasmid+0.1 µg NLRP3 expression construct, or control vector was introduced under the application of Lipofectamine 3000 (#L3000150, Invitrogen, Bremen, Germany).

### mRNA analysis of NLRP3 and FMR1

BV2 cells were subjected to LPS/ATP exposure, MCC950 + LPS/ATP co-treatment, FMR1 plasmid or vector control transaction before LPS/ATP exposure, or vehicle mock, and followed by preparation of cellular total RNA using TRIzol (#15596026, Invitrogen). cDNA was oligo(dT) primed from 500 ng of RNA using M-MLV Reverse Transcriptase as described (#28025013, Invitrogen). The qPCR mixture for a reaction consisted of 1 µL cDNA template, 8.2 µL nuclease-free water, 0.4 µL of each primer (NLRP3-sense: 5’-GACCGTGAGGAAAGGACCAG-3’ and NLRP3-antisense: 5’-GGCCAAAGAGGAATCGGACA-3’; FMR1-sense: 5’-AACGACGATCATTCCCGAACA-3’ and FMR1-antisense: 5’-GGGTACTCCATTCACCAGCG-3’;), and 10 µL SYBR Green Mix (Servicebio). Data were plotted as fold change (2^-ΔΔCt^) after normalization to a reference GAPDH (sense: 5’-TGGAAAGCTGTGGCGTGAT-3’ and antisense: 5’-GTTGCTGTTGAAGTCGCAGG-3’).

### Western blot

BV2 cells were subjected to LPS/ATP exposure, MCC950 + LPS/ATP co-treatment, or vehicle mock treatment, or the relevant transaction before LPS/ATP exposure, which was followed by protein isolation in cold RIPA buffer (#P0013B, Beyotime) in the presence of a cocktail of protease and phosphatase inhibitors (#78442, Thermo Fisher Scientific, Milan, Italy). After SDS-PAGE, the resulting gels were blotted (100 V; 60–70 min) to nitrocellulose by wet transfer. For immunoblot analyses, we employed anti-NLRP3 mouse pAb (#GB114320, 1:3000, Servicebio), anti-caspase-1 rabbit pAb (#ab286125, 1:200, Abcam), anti-FMR1 rabbit pAb (#13755–1-AP, 1:2000, Proteintech), anti-IGF2 BP3 rabbit pAb (#14642–1-AP, 1:30000, Proteintech), anti-IL-1β goat pAb (#AF-401-NA, 1:8000, R&D Systems), and anti-β-actin mouse mAb (#ab8226, 1:1000, Abcam). Anti-rabbit (#ab6721, 1:1000, Abcam) and anti-mouse (#HRP-67762, 1:5000, Proteintech) secondary antibodies were labeled by HRP. For HRP detection, the BeyoECL Plus Kit (#P0018S) was applied as described by the vendor (Beyotime). The original images of WB assay can be found in S1 File.

### Measurement of caspase-1 activity

BV2 cells were subjected to LPS/ATP exposure, MCC950 + LPS/ATP co-treatment, or vehicle mock treatment, or the indicated plasmid transaction (vector, FMR1, or FMR1 + NLRP3) before LPS/ATP exposure. After that, cells were obtained for caspase-1 activity detection with Caspase-1 Activity Assay Kit (#C1102, Beyotime) as per the accompanying guidelines.

### Determination of secretion levels of IL-1β and IL-18

BV2 cells were subjected to the indicated treatment (LPS/ATP, MCC950 + LPS/ATP, or vehicle) or transaction (vector, FMR1, or FMR1 + NLRP3) prior to LPS/ATP treatment. The levels of IL-1β and IL-18 in the supernatant of treated BV2 microglia were determined using Mouse IL-1β ELISA Kit (#EK201B) and Mouse IL-18 ELISA Kit (#EK218), respectively, as described by the vendor (Multi Sciences, Hangzhou, China). The Infinite M200 ELISA reader (Tecan, Männedorf, Switzerland) was utilized for absorbance measurement at 450 nm.

### RNA pull-down assay and mass spectrometry (MS)

To study the interactors of NLRP3 3’UTR, we performed RNA pull-down assay under the use of biotinylated mmu_NLRP3 3’UTR probes and the Pure Magnetic RNA-Protein Pull-down Kit (#RY6003, Writegene Biotechnology Co., Ltd., Zhengzhou, China). The biotin labeling efficiency of mmu_NLRP3 3’UTR probes was confirmed by the efficiency assay using Biotin-labeling efficiency Detection Kit (#RY3011) and protocols (Writegene Biotechnology Co., Ltd.). Following the Pull-down Kit instructions, the complex of magnetic bead and probe was firstly generated by adding biotinylated mmu_NLRP3 3’UTR probes into the pre-treated magnetic beads. Lysates of LPS/ATP-challenged BV2 microglia were then added to the complex of magnetic bead and probe and applied for 1 h at room temperature. The precipitated complex was harvested, and bound proteins were eluted with the mixture of 80 µL 1 × Washing buffer and 20 µL 5 × Loading buffer by boiling for 10 min.

The precipitated proteins were processed by MS analysis by Qinglianbio Biotechnology Co., Ltd. (Beijing, China) or immunoblot analysis for FMR1 enrichment evaluation. Through RIGOL L-3000 HPLC System (RIGOL, Beijing, China), MS analysis was carried out and subsequently analyzed using Proteome Discoverer2.4 software from Thermo Fisher Scientific.

### Luciferase assay

The NLRP3 3’UTR, amplified by PCR from mouse genomic DNA, was inserted into pmirGLO Dual-Reporter vector (#VT1439, YouBio, Changsha, Hunan) via appropriate sites. Utilizing Lipofectamine 3000, the generated reporter construct along with or without FMR1 cDNA plasmid or control vector was introduced into BV2 microglia. Forty-eight hours post-transfection, cells were subjected to luciferase activity analysis under the use of Dual-Lumi Reporter Kit (#G1701, Servicebio).

### Analysis of RNA stability

BV2 cells transfected with or without FMR1 cDNA plasmid or control vector were performed with LPS/ATP exposure or control treatment. Actinomycin D (99.74% purity, #S8964, Selleck) diluted in DMSO was then added into the medium at the 10 µg/mL final concentration and applied for 0, 1, 2, 3, 4, or 5 h at 37°C. Cells were obtained and assayed for NLRP3 mRNA level by qPCR analysis.

### Lactate dehydrogenase (LDH) release assay

LDH activity in the cell culture supernatant was measured as an indicator of cytotoxicity and pyroptosis using a colorimetric LDH assay kit (#A020-2, Jiancheng Bioengineering Institute). BV2 cells were seeded in 96-well plates and subjected to the indicated treatments or transfections. Following stimulation with LPS and ATP, the culture supernatants were collected by centrifugation at 400 × g for 5 min at room temperature. The LDH activity in the supernatant was determined according to the manufacturer’s instructions. In brief, 20 μL of supernatant was mixed with the working solution containing lactate, NAD^+^, and INT, followed by incubation at 37°C for 30 min. The reaction was stopped by adding the stop solution, and the absorbance at 450 nm was measured using a microplate reader (MULTISKAN MK3, Thermo Scientific). The LDH activity was calculated based on a standard curve generated with a pyruvate standard and normalized to the total LDH activity released from fully lysed cells.

### Calcein-AM/PI staining assay

Cell viability and cytotoxicity were assessed using a Calcein-AM/PI double staining assay. Following the indicated transfections and treatments, BV2 cells cultured in 96-well plates were washed once with PBS. A working solution was prepared by diluting Calcein-AM (1000×) and Propidium Iodide (PI, 1000×) from a commercial kit (#C2015L, Beyotime) to 1× in serum-free medium. Cells were incubated with this staining solution in the dark at 37°C for 20 min. After incubation, fluorescent images were captured using a fluorescence microscope (OLYMPUS CKX53). Viable cells exhibiting green fluorescence (Calcein-AM positive) and dead cells exhibiting red nuclear fluorescence (PI positive) were counted manually or using image analysis software (ImageJ, NIH), and the percentage of viable cells was calculated.

### Lentiviral delivery of FMR1 in EAE mice

To investigate the therapeutic effect of FMR1 *in vivo*, EAE mice were randomly divided into three groups (n = 6/group): EAE, EAE + Lv-con (control lentivirus), and EAE + Lv-FMR1 (FMR1-overexpressing lentivirus). All procedures were approved by the Animal Ethics Committee of the First Affiliated Hospital of Henan University of Science and Technology. Lentiviral particles (1 × 10⁸ TU/mouse) were intrathecally injected into the lumbar spinal cord at day 7 post-immunization under anesthesia with 3% isoflurane to minimize pain. The lentiviral vectors (GenePharma, China) were constructed by cloning the full-length mouse FMR1 cDNA into the pLVX-Puro vector. Buprenorphine (0.1 mg/kg) was subcutaneously administered every 12 h for 24 h to alleviate pain. Mice were monitored daily for clinical scores, weight, and abnormalities. Mice with scores ≥3 received soft food/hydration gels. At the endpoint, mice were euthanized via intraperitoneal injection of sodium pentobarbital (150 mg/kg).

### Statistics and bioinformatics

Graphpad Prism 8.0 was used for data analysis. Normality was assessed via Shapiro-Wilk test (α = 0.05), and homogeneity of variance was confirmed by Levene’s test. All data meet normality and homogeneity of variance, and were represented as the mean±standard deviation. Significance was defined as < 0.05 by using an unpaired *t*-*t*est (two groups) or one-way ANOVA (three groups). Under the use of clusterProfiler in R package (4.2.1), the GO enrichment analysis of these precipitated proteins after MS analysis was conducted.

## Results

### Activation of microglia and upregulation of NLRP3 in the spinal cord of EAE mice

**Fig 1:**
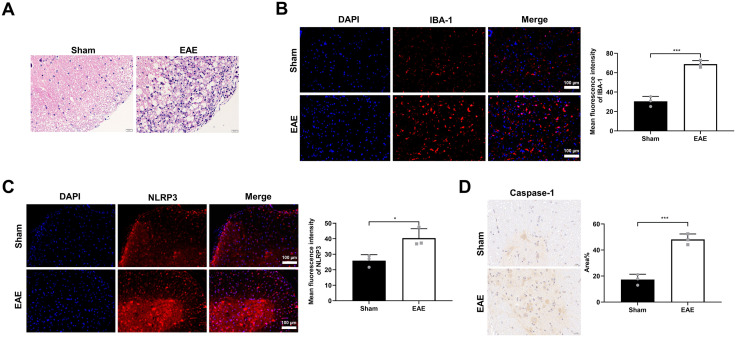
NLRP3 upregulation and microglia activation in the spinal cord of EAE mice. **(A)** Representative H&E staining of spinal cord tissues from Sham and EAE mice. **(B and C)** Representative immunofluorescence images and quantification depicting the expression of IBA-1 (B) and NLRP3 (C) in spinal cord tissues. Nuclei were counterstained with DAPI (blue). Scale bar, 100 µm. Data are expressed as mean ± SD of 3 biologically independent experiments. **(D)** Representative immunohistochemical staining and quantification of caspase-1 in spinal cord tissues. Data represent mean ± SD from 3 randomly selected mice per group (n = 6 biological replicates per group). *P < 0.05, ***P < 0.001.

The NLRP3 inflammasome is a well-established contributor to MS pathology [4]. In this study, we employed the experimental autoimmune EAE model, induced by MOG35–55/CFA immunization in female C57BL/6 mice, to recapitulate key neuroinflammatory features of MS [23,25]. Histopathological analysis revealed distinct inflammatory cell infiltration in the spinal cords of EAE mice compared to Sham controls (Fig 1A), confirming successful model establishment. Given the critical role of microglial dysregulation in MS progression [2], we assessed microglial activation. Immunofluorescence staining demonstrated a significant increase in the expression of the microglia marker IBA-1 in EAE mice (68.87 ± 3.65) compared to sham controls (30.53 ± 5.09) controls (Fig 1B; R^2^ = 0.97, 95% confidence interval = 28.30 ～ 48.37), indicating robust microglia activation. Concurrently, NLRP3 expression was markedly elevated in the spinal cords of EAE mice (40.45 ± 6.05) relative to sham controls (25.91 ± 3.81) controls (Fig 1C; R^2^ = 0.76, 95% confidence interval = 3.09 ～ 26.00). As caspase-1 is a key effector component activated by the NLRP3 inflammasome [3], we next examined its expression. Immunohistochemical analysis confirmed a substantial increase in caspase-1 deposition in the spinal cords of EAE mice (48.16 ± 4.18) compared to sham controls (17.33 ± 4.10) (Fig 1D; R^2^ = 0.95, 95% confidence interval = 21.45 ～ 40.22). Collectively, these results demonstrate that NLRP3 inflammasome activation is associated with neuroinflammation in the EAE model of MS.

### LPS/ATP activates the NLRP3 inflammasome in BV2 microglia

**Fig 2:**
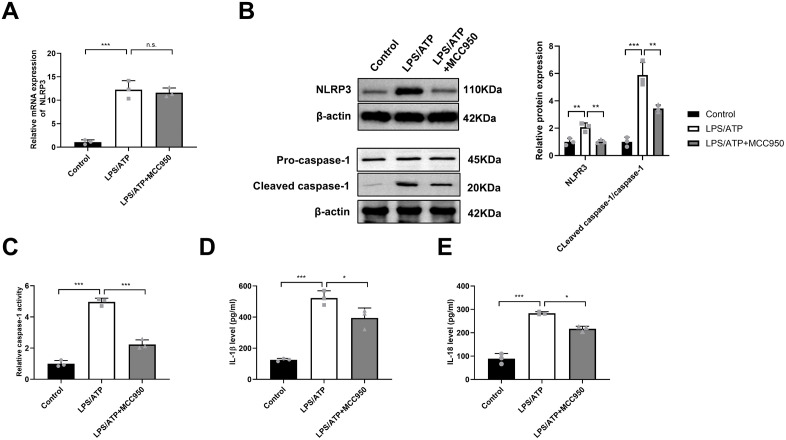
LPS/ATP activates the NLRP3 inflammasome in BV2 microglia. **(A)** qPCR analysis of NLRP3 mRNA in BV2 cells treated with vehicle (PBS), LPS/ATP, or LPS/ATP + MCC950. Data are expressed as mean ± SD of 3 biologically independent experiments. **(B)** Representative Western blots and quantification of NLRP3, pro-caspase-1, and cleaved caspase-1 (p20) protein levels. β-actin served as a loading control. Data are expressed as mean ± SD of 3 biologically independent experiments. **(C)** Caspase-1 activity measured using a commercial assay kit. Data are expressed as mean ± SD of 3 biologically independent experiments. **(D and E)** Secretion levels of mature IL-1β and IL-18 in cell culture supernatants determined by ELISA. Data are expressed as mean ± SD of 3 biologically independent experiments. *P < 0.05, **P < 0.01, ***P < 0.001, n.s. non-significant.

The LPS/ATP stimulation paradigm is widely used to model NLRP3 inflammasome activation in microglia [26,27]. In BV2 microglial cells, LPS/ATP exposure significantly increased both NLRP3 mRNA (Fig 2A) and protein levels (Fig 2B). Co-treatment with the specific NLRP3 inhibitor MCC950 did not affect NLRP3 transcription (Fig 2A) but reduced NLRP3 protein abundance (Fig 2B), suggesting potential post-transcriptional regulation. LPS/ATP stimulation dramatically promoted caspase-1 activity, as evidenced by increased levels of cleaved caspase-1 (p20) (Fig 2B) and enhanced enzymatic activity (Fig 2C). This activation was effectively inhibited by MCC950 co-treatment (Fig 2B and 2C). Consistent with inflammasome activation, ELISA revealed elevated secretion of the mature cytokines IL-1β and IL-18 in LPS/ATP-treated BV2 cells, both of which were suppressed by MCC950 (Fig 2D and 2E). These results align with prior evidence [9,28] and confirm robust NLRP3 inflammasome activation in our cellular model.

### FMR1 interacts with the NLRP3 3’UTR in LPS/ATP-stimulated BV2 microglia

**Fig 3:**
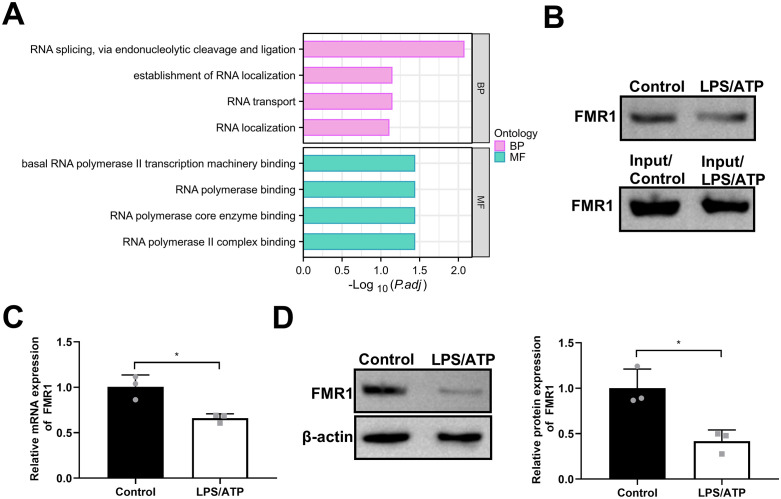
The RBP FMR1 interacts with the NLRP3 3’UTR. **(A)** GO enrichment analysis of proteins pulled down by biotinylated NLRP3 3’UTR probes in LPS/ATP-stimulated BV2 cells. **(B)** Western blot analysis confirming the interaction between FMR1 and the NLRP3 3’UTR. Cell lysates (Input) and proteins pulled down by the biotinylated probe (Biotin Pull-down) were probed with an anti-FMR1 antibody. **(C and D)** qPCR **(C)** and western blot **(D)** analysis showing downregulation of FMR1 mRNA and protein expression in BV2 cells after LPS/ATP stimulation. Data are expressed as mean ± SD of 3 biologically independent experiments. *P < 0.05.

Given the therapeutic potential of targeting NLRP3 [11], we sought to identify its post-transcriptional regulators. We performed an RNA pull-down assay using biotinylated NLRP3 3’UTR probes in LPS/ATP-treated BV2 cells followed by mass spectrometry, which identified 2214 potential interacting proteins (S1 Table). GO enrichment analysis of these proteins revealed strong association with RNA-related processes, including RNA localization, transport, and polymerase binding (Fig 3A). By cross-referencing these interactors with potential NLRP3-binding RBPs predicted by the POSTAR3 database, we selected FMR1 for further investigation due to its established neurological functions [18]. The interaction between FMR1 protein and the NLRP3 3’UTR was validated by Western blot analysis of the RNA pull-down precipitates (Fig 3B). Notably, LPS/ATP stimulation reduced the amount of FMR1 protein associated with the NLRP3 3’UTR probe (Fig 3B). We subsequently examined FMR1 expression in BV2 cells and found that LPS/ATP stimulation significantly decreased both FMR1 mRNA and protein levels (Fig 3C and 3D). These data indicate that FMR1 interacts with the NLRP3 3’UTR and that its expression is suppressed under inflammatory conditions.

### FMR1 destabilizes NLRP3 mRNA to suppress its expression

**Fig 4:**
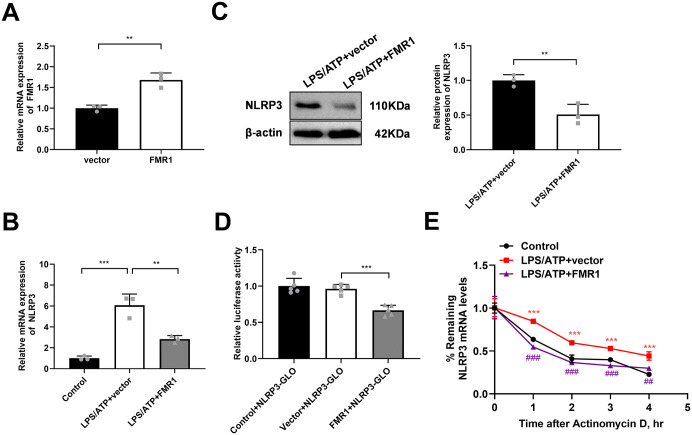
FMR1 destabilizes NLRP3 mRNA in LPS/ATP-challenged BV2 microglia. **(A)** qPCR confirmation of FMR1 overexpression in transfected BV2 cells. Data are expressed as mean ± SD of 3 biologically independent experiments. **(B and C)** NLRP3 mRNA and protein expression in BV2 cells overexpressing FMR1 or vector control upon LPS/ATP stimulation. Data are expressed as mean ± SD of 3 biologically independent experiments. **(D)** Luciferase activity of an NLRP3 3’UTR reporter construct in BV2 cells co-transfected with FMR1 or control vector. Data are expressed as mean ± SD of 5 biologically independent experiments. **(E)** NLRP3 mRNA stability assessed by actinomycin D chase assay in BV2 cells overexpressing FMR1 or vector control after LPS/ATP treatment. Data are expressed as mean ± SD of 3 biologically independent experiments. **P < 0.01, ***P < 0.001.

Having established the FMR1-NLRP3 3’UTR interaction, we investigated its functional consequence. Overexpression of FMR1 in BV2 cells (Fig 4A) led to a significant decrease in NLRP3 expression at both the mRNA (Fig 4B) and protein levels (Fig 4C) upon LPS/ATP stimulation, indicating that FMR1 acts as a negative regulator of NLRP3. To rule out the possibility that this reduction was an indirect effect of caspase-1-mediated feedback cleavage, we used the pan-caspase inhibitor z-VAD. As shown in S1 Fig, z-VAD pretreatment effectively abrogated the cleavage of caspase-1 and the maturation of IL-1β, confirming the complete inhibition of caspase-1 activity. Notably, under this condition, the protein level of NLRP3 in the z-VAD+Vector control group was comparable to that in the DMSO+Vector group, arguing against a significant role for caspase-1-mediated cleavage in regulating NLRP3 protein turnover. Most importantly, FMR1 overexpression still significantly reduced NLRP3 protein levels in the presence of z-VAD (z-VAD + FMR1 group vs. z-VAD+Vector group). This result provides compelling evidence that the downregulation of NLRP3 by FMR1 is not a secondary consequence of caspase-1 activation but is a primary event that occurs upstream and independently of the inflammasome effector functions. A luciferase reporter assay containing the NLRP3 3’UTR showed that FMR1 overexpression suppressed its activity (Fig 4D), indicating direct regulation at the post-transcriptional level. Furthermore, an mRNA stability assay using actinomycin D demonstrated that FMR1 overexpression accelerated the decay of NLRP3 mRNA (Fig 4E). These results collectively demonstrate that FMR1 acts as a post-transcriptional inhibitor of NLRP3 by binding to its 3’UTR and promoting mRNA degradation.

Our RNA pull-down/MS screen also identified IGF2 BP3, a known stabilizing RBP, as a binding partner of the NLRP3 3’UTR (S1 Table). We hypothesized that FMR1 and IGF2 BP3 might compete for binding. To test this, we performed a competitive RNA pull-down assay. Overexpression of FMR1 in BV2 cells significantly reduced the binding of IGF2 BP3 to the biotinylated NLRP3 3’UTR probe. Conversely, overexpression of IGF2 BP3 diminished the association of FMR1 with the NLRP3 mRNA (S2 Fig). These results demonstrate that FMR1 and IGF2 BP3 compete for binding to the NLRP3 3’UTR, suggesting that the downregulation of FMR1 under inflammatory conditions may shift the balance towards IGF2 BP3-mediated stabilization, thereby promoting NLRP3 expression.

### FMR1 suppresses NLRP3 inflammasome activation by downregulating NLRP3

**Fig 5:**
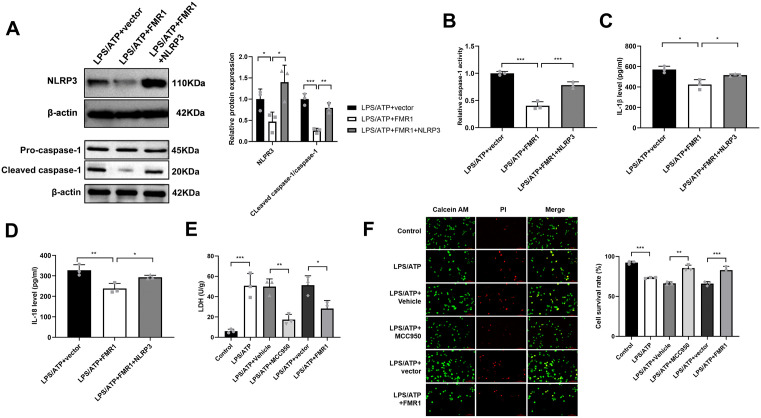
FMR1 suppresses NLRP3 inflammasome activation and pyroptosis by downregulating NLRP3. **(A)** Measurement of NLRP3, pro-caspase-1 and cleaved caspase-1 levels by western blot in BV2 cells transfected with FMR1 expression construct, FMR1 expression construct+NLRP3 cDNA plasmid, or vector control before LPS/ATP exposure. Data are expressed as mean ± SD of 3 biologically independent experiments. **(B)** Evaluation of caspase-1 activity in BV2 cells treated as indicated using the assay kit. Data are expressed as mean ± SD of 3 biologically independent experiments. **(C and D)** Determination of IL-1β and IL-18 secretion levels by ELISA in the supernatant of BV2 cells treated as in A. Data are expressed as mean ± SD of 3 biologically independent experiments. **(E)** BV2 cells were transfected and treated as indicated, followed by LPS/ATP stimulation. The release of LDH into the culture supernatant was quantified as a marker of cell membrane integrity loss during pyroptosis. Data are expressed as mean ± SD of 3 biologically independent experiments. **(F)** FMR1 attenuates LPS/ATP-induced pyroptosis, as assessed by Calcein-AM/PI staining. Representative fluorescent micrographs (left) and quantification data (right). Nuclei were counterstained with DAPI (blue). Scale bar, 100 μm. Data are expressed as mean ± SD of 3 biologically independent experiments. *P < 0.05, **P < 0.01, ***P < 0.001.

We next investigated the functional impact of FMR1-mediated NLRP3 downregulation on inflammasome activity. In LPS/ATP-stimulated BV2 cells, FMR1 overexpression significantly reduced the levels of cleaved caspase-1 (p20) (Fig 5A) and caspase-1 enzymatic activity (Fig 5B). Consequently, the secretion of the mature cytokines IL-1β and IL-18 was also attenuated (Fig 5C and 5D). Crucially, co-expression of NLRP3 cDNA alongside FMR1 rescued these inhibitory effects (Fig 5A–5D), confirming that FMR1 suppresses inflammasome activation specifically through downregulation of NLRP3.

The ultimate functional outcome of NLRP3 inflammasome activation is the induction of pyroptosis, a lytic and inflammatory form of programmed cell death. To determine whether FMR1-mediated suppression of the inflammasome translates to this critical pathological event, we measured pyroptosis using LDH release assay and Calcein-AM/PI staining. LPS/ATP stimulation triggered a robust increase in LDH release (Fig 5E) and reduced cell survival rate (Fig 5F), indicative of extensive pyroptotic cell death. This effect was abolished by the NLRP3 inhibitor MCC950, confirming the specificity of the response. Crucially, overexpression of FMR1 significantly attenuated LPS/ATP-induced LDH release and improved cell survival. These data demonstrate that FMR1 not only inhibits the molecular assembly and cytokine production of the NLRP3 inflammasome but also functionally blocks its downstream pyroptotic cell death pathway.

### FMR1 overexpression alleviates neuroinflammation in EAE mice

**Fig 6:**
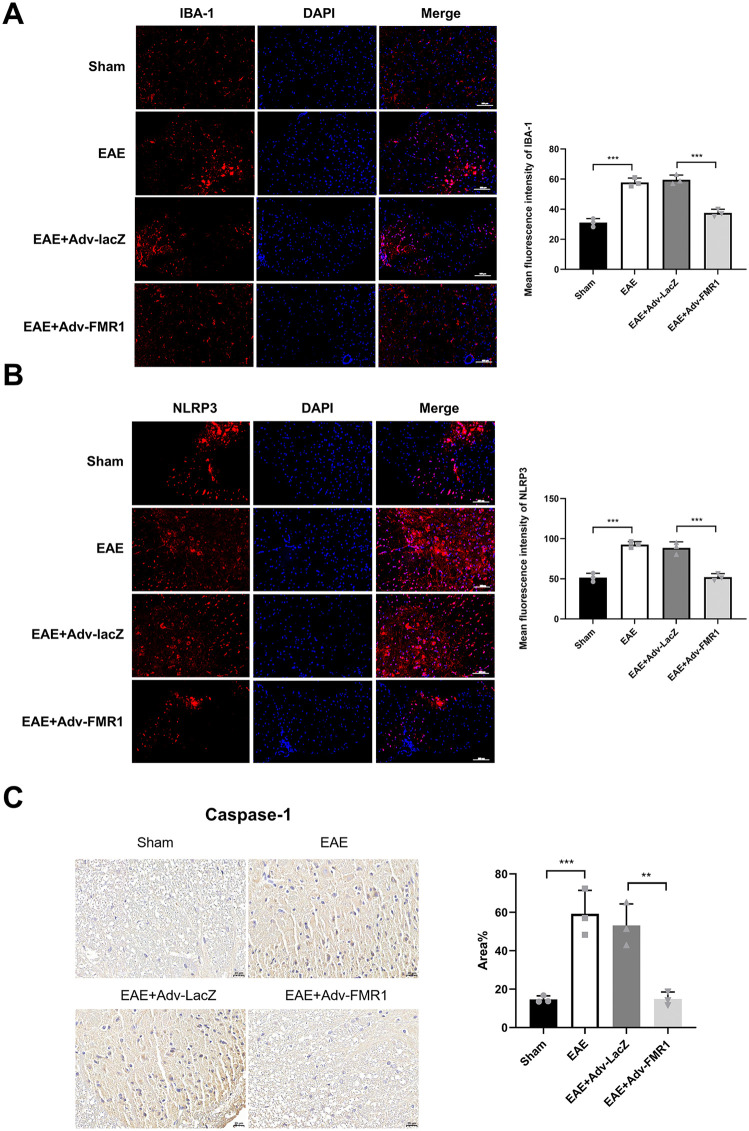
FMR1 overexpression attenuates NLRP3 inflammasome activation and neuroinflammation in EAE mice. **(A and B)** Representative immunofluorescence images of IBA-1 (A) and NLRP3 (B) in spinal cord tissues from Sham, EAE, EAE + Lv-con, and EAE + Lv-FMR1 mice. Nuclei were counterstained with DAPI (blue). Data are expressed as mean ± SD of 3 biologically independent experiments. **(C)** Representative immunohistochemical images of caspase-1 deposition in spinal cord tissues. Data represent mean ± SD from 3 randomly selected mice per group (n = 6 biological replicates per group). **P < 0.01, ***P < 0.001.

To validate the therapeutic potential of FMR1 *in vivo*, we overexpressed FMR1 in EAE mice via lentiviral delivery (EAE + Lv-FMR1). Immunofluorescence analysis of spinal cord tissues revealed that EAE mice exhibited robust microglial activation (IBA-1) and elevated NLRP3 expression compared to Sham controls (IBA-1: 57.82 ± 2.91 vs 31.02 ± 2.76; NLRP3: 92.71 ± 3.74 vs 51.62 ± 5.29). FMR1 overexpression significantly attenuated these increases (Fig 6A and 6B; IBA-1: 37.48 ± 2.47 vs 59.56 ± 3.13; NLRP3: 52.10 ± 4.31 vs 88.50 ± 7.53). Similarly, immunohistochemical staining demonstrated that caspase-1 deposition was markedly reduced in the EAE + Lv-FMR1 group (14.89 ± 3.62) compared to the EAE + Lv-con group (53.14 ± 11.22) (Fig 6C). These *in vivo* findings confirm that FMR1 suppresses NLRP3 inflammasome activation and neuroinflammation, aligning with our cellular findings and supporting FMR1 as a potential therapeutic target for MS.

## Discussion

The NLRP3 inflammasome is a key driver of pathology in a broad spectrum of inflammatory disorders, including Alzheimer’s disease, arthritis, and cryopyrin-associated periodic syndrome [29]. Its critical role in the pathogenesis of MS is increasingly recognized [4], spurring interest in identifying novel strategies to inactivate this inflammasome. Here, we identify the RBP FMR1 as a previously unrecognized post-transcriptional regulator that destabilizes NLRP3 mRNA and suppresses inflammasome activation in microglial models of neuroinflammation. Our findings suggest that targeting the FMR1-NLRP3 axis may represent a promising therapeutic strategy for MS.

Consistent with prior studies [9,30,31], we confirmed robust activation of the NLRP3 inflammasome in the spinal cords of EAE mice and in LPS/ATP-stimulated BV2 microglia. Notably, pharmacologic inhibition with MCC950—a selective NLRP3 inhibitor [24]— reduced levels of cleaved caspase-1 and secretion of IL-1β/IL-18 without affecting NLRP3 transcription, pointing to a post-transcriptional mode of NLRP3 regulation. Intriguingly, MCC950 similarly downregulates NLRP3 protein (but not mRNA) in models of atherosclerotic and diabetic stroke [32,33], suggesting a conserved regulatory mechanism across inflammasome-associated diseases.

RBPs are master regulators of post-transcriptional gene expression, modulating processes such as mRNA stability through sequence-specific binding [34]. Several RBPs have been reported to regulate NLRP3 mRNA stability. For instance, HuR stabilizes NLRP3 mRNA via direct interaction with AU-rich elements in its 3’UTR, contributing to cerebral ischemic stroke [13]. METTL3, an m6A methyltransferase, enhances NLRP3 mRNA stability in BPDE-treated trophoblast cells, thereby promoting inflammasome activation and pyroptosis [14]. Furthermore, the m6A reader IGF2 BP3 stabilizes NLRP3 mRNA in a WTAP-dependent manner, activating the NLRP3 inflammasome and pro-inflammatory responses [12]. However, negative RBP regulators of NLRP3 mRNA have remained elusive.

FMR1 premutation or loss-of-function is implicated in various disorders, including ovarian insufficiency, sleep apnea, and neuropathy [15]. In inflammation, FMR1 mediates the loading and secretion of miRNA-containing exosomes [35], and its depletion attenuates pro-inflammatory responses in LPS/ATP-exposed microglia [17]. Aberrant FMR1 expression also contributes to several neurological conditions, such as fragile X tremor ataxia syndrome [15], amyotrophic lateral sclerosis [36], and MS [18,19]. Our study reveals a direct interaction between FMR1 and the NLRP3 3’UTR in LPS/ATP-challenged BV2 microglia. A key finding is that FMR1 suppresses NLRP3 mRNA stability. Our competitive pull-down data provide direct evidence that FMR1 and IGF2 BP3 antagonistically bind to the NLRP3 3’UTR. This competitive interaction offers a plausible mechanism for the precise control of NLRP3 mRNA stability. Future studies employing CLIP-seq will be essential to map the exact binding sites and determine whether this competition is direct or allosteric. In line with our findings, a recent study demonstrated that FMR1 promotes the degradation of RAC1 mRNA during melanoma metastasis [37]. Conversely, FMR1 has also been reported to stabilize mRNAs such as EGFR in colorectal tumorigenesis [38] and PDHA1 in prostate cancer development [39], highlighting its context-dependent functions. Most importantly, we identify FMR1 as a potent suppressor of the NLRP3 inflammasome in microglia, exerting its effect through the downregulation of NLRP3. Taken together, our findings demonstrate that FMR1 diminishes NLRP3 expression, at least in part, by destabilizing its mRNA, thereby inhibiting inflammasome activation.

The clinical relevance of FMR1 is underscored by its ability to mitigate neuroinflammation in EAE mice. Consistent with our *in vitro* results, lentiviral overexpression of FMR1 in EAE mice significantly reduced microglial activation (IBA-1), NLRP3 expression, and caspase-1 activity in the spinal cord. These *in vivo* results highlight the translational potential of FMR1 as a therapeutic target for MS. Lentiviral delivery of FMR1 not only validated its anti-inflammatory role in a disease model but also bridged the gap between cellular mechanism and complex pathophysiology. The reduction in NLRP3 and caspase-1 levels in FMR1-overexpressing mice indicates that FMR1-mediated mRNA destabilization translates to functional suppression of inflammasome activity *in vivo*.

Despite these advances, our study has limitations. while we focused on NLRP3, FMR1 may regulate other inflammatory mediators (e.g., IL-6 or TNF-α) in microglia, warranting further investigation. Additionally, given that FMR1 can suppress the translation of some target mRNAs [40,41], future studies should explore whether it also represses NLRP3 protein synthesis independently of mRNA decay.

**Fig 7:**
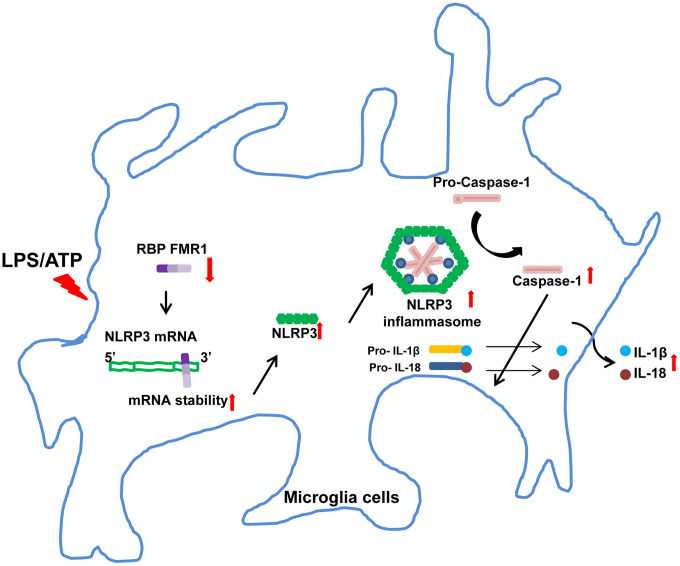
Schematic diagram of the RBP FMR1-NLRP3 inflammasome cascade in LPS/ATP-challenged BV2 microglia. In LPS/ATP-challenged BV2 microglia, FMR1 mediates NLRP3 mRNA stability and thus regulates NLRP3 expression, thereby affecting the activation of the NLRP3 inflammasome.

In conclusion, our findings establish the RBP FMR1 as a critical post-transcriptional suppressor of the NLRP3 inflammasome in both cellular and animal models of neuroinflammation (Fig 7). By destabilizing NLRP3 mRNA, FMR1 potently inhibits inflammasome activation and downstream pyroptosis. Enhancing FMR1 activity or expression may therefore represent a novel therapeutic strategy to mitigate neuroinflammation in multiple sclerosis and related neurodegenerative disorders.

## Supporting information

S1 Fig FMR1-mediated downregulation of NLRP3 is independent of caspase-1 activity. BV2 cells were pretreated with DMSO vehicle or the pan-caspase inhibitor z-VAD (20 µM) for 2 h, followed by transaction with vector control or FMR1-overexpressing plasmid. After 24 h, all groups were stimulated with LPS/ATP to activate the NLRP3 inflammasome. Representative Western blots and quantitative analysis of protein levels of NLRP3, pro-caspase-1, cleaved caspase-1 (p20), pro-IL-1β, and mature IL-1β (p17) in BV2 cells treated as in (A). β-actin served as a loading control. *P < 0.05, **P < 0.01, ***P < 0.001.
(TIF)

S2 Fig FMR1 and IGF2 BP3 competitively bind to the NLRP3 3’UTR. Competitive RNA pull-down assay in BV2 microglial cells transfected with vector control, FMR1-, or IGF2 BP3-overexpressing plasmids, using a biotin-labeled NLRP3 3’UTR probe.
(TIF)

S1 Table The 2214 precipitated proteins pulled down by biotin-labeled NLRP3 3’UTR probes in LPS-challenged BV2 microglia. (XLSX)

S1 File Raw images of WB assay. (PDF)
