# Supplementary figures and images for: A new mechanism regulating microglial NLRP3 inflammasome: FMR1 mediates NLRP3 mRNA stability
Source: PLoS One. 2026 Feb 20;21(2):e0341867. doi: 10.1371/journal.pone.0341867 (PMC12922985; doi:10.1371/journal.pone.0341867)

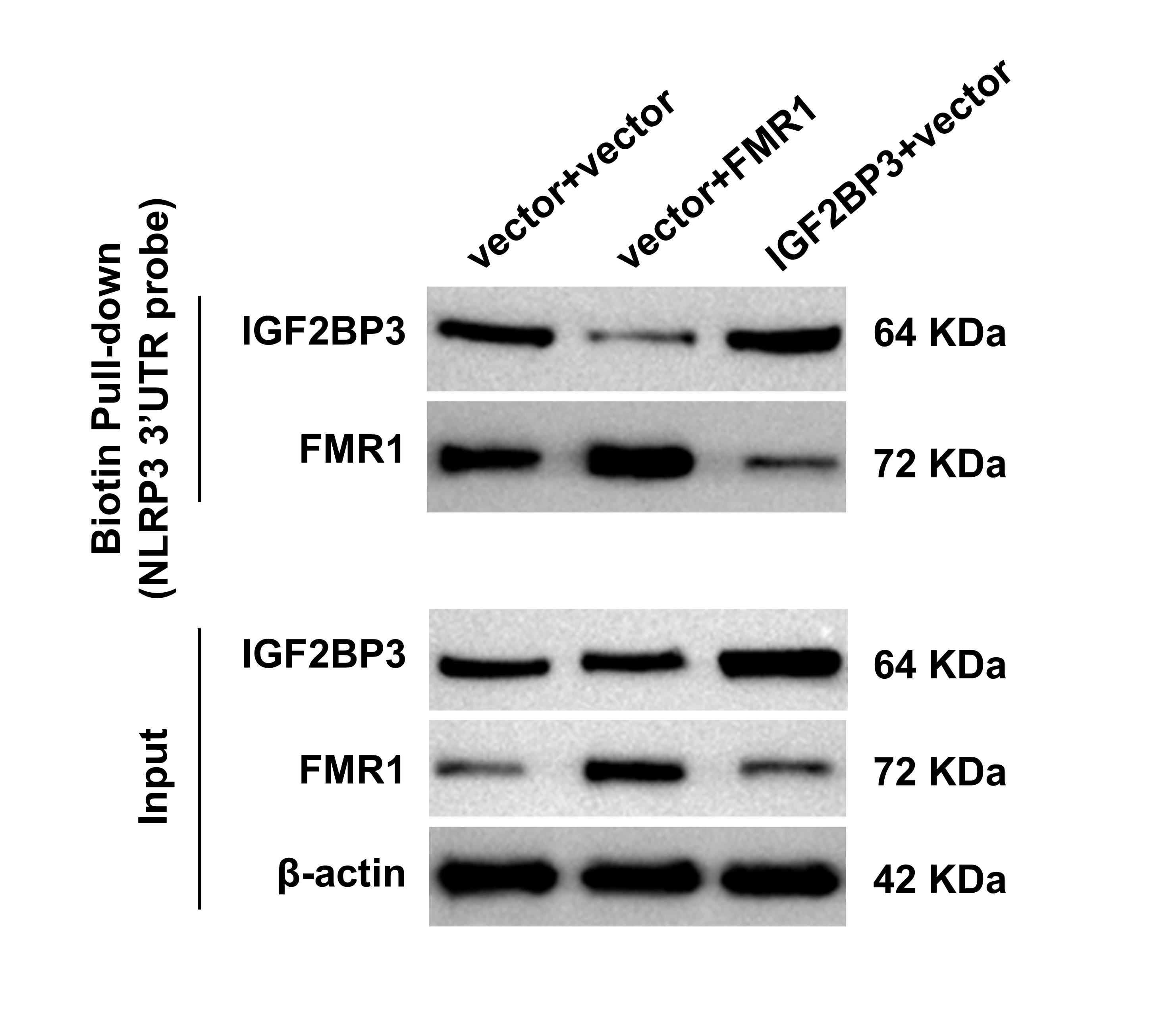

Supplement: S2 Fig — Competitive RNA pull-down assay in BV2 microglial cells transfected with vector control, FMR1-, or IGF2 BP3-overexpressing plasmids, using a biotin-labeled NLRP3 3’UTR probe. (TIF) [file pone.0341867.s002.tif]
