# Supplementary material for: A new mechanism regulating microglial NLRP3 inflammasome: FMR1 mediates NLRP3 mRNA stability
Source: PLoS One. 2026 Feb 20;21(2):e0341867. doi: 10.1371/journal.pone.0341867 (PMC12922985; doi:10.1371/journal.pone.0341867)
Supplement: S1 File — (PDF) [file pone.0341867.s004.pdf]

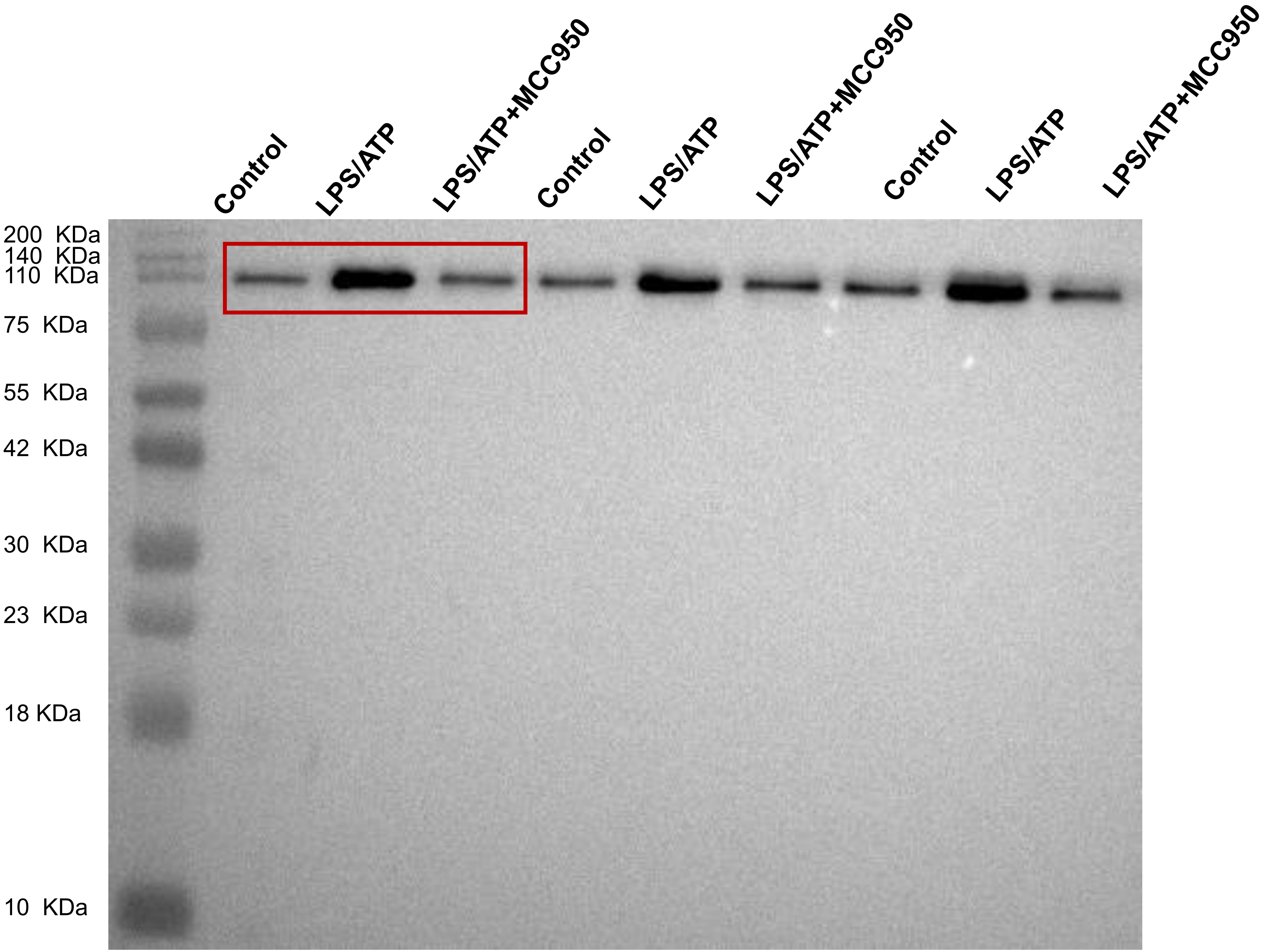

Figure 2B: NLRP3 (110 KDa)

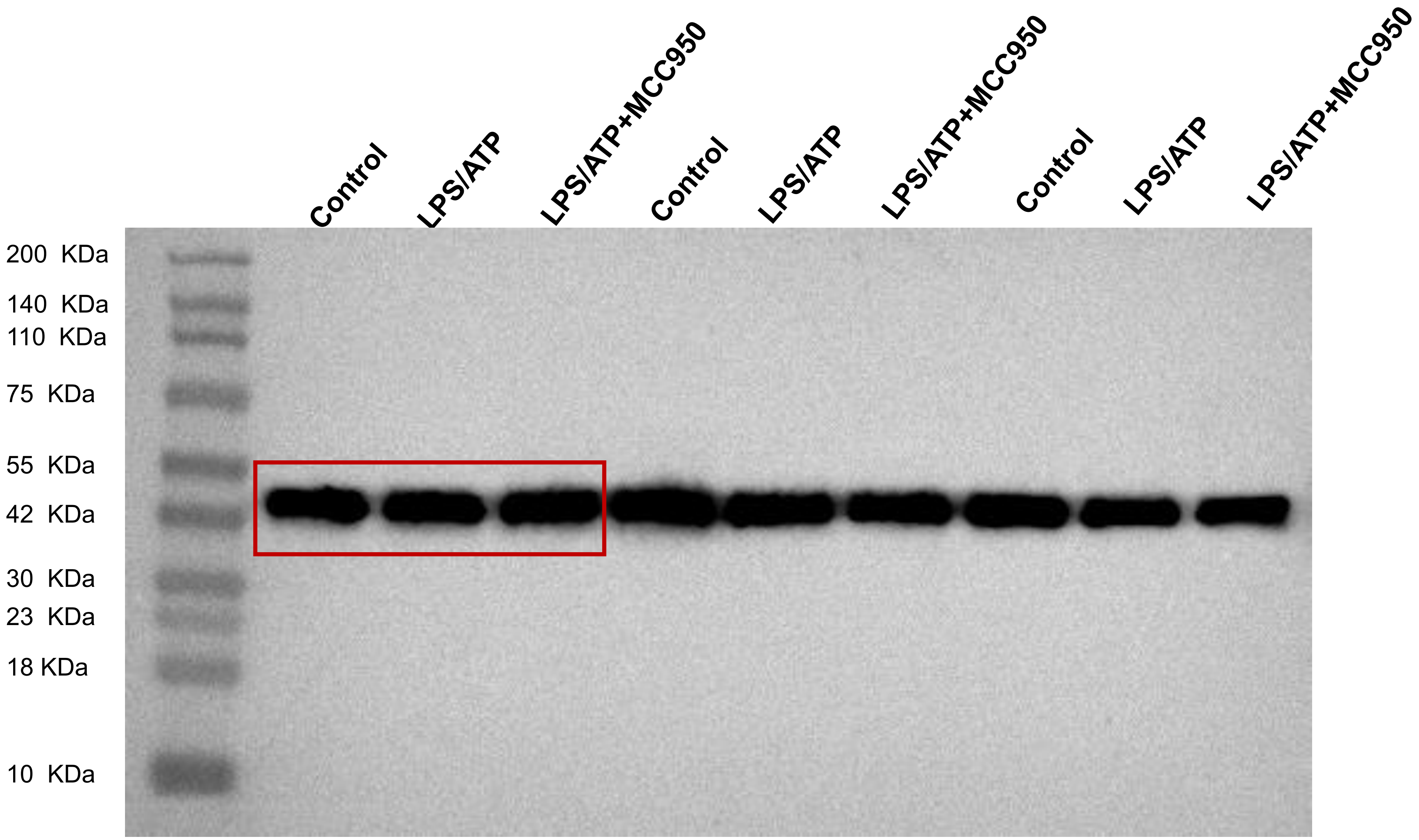

Figure 2B: β-actin (42 KDa)

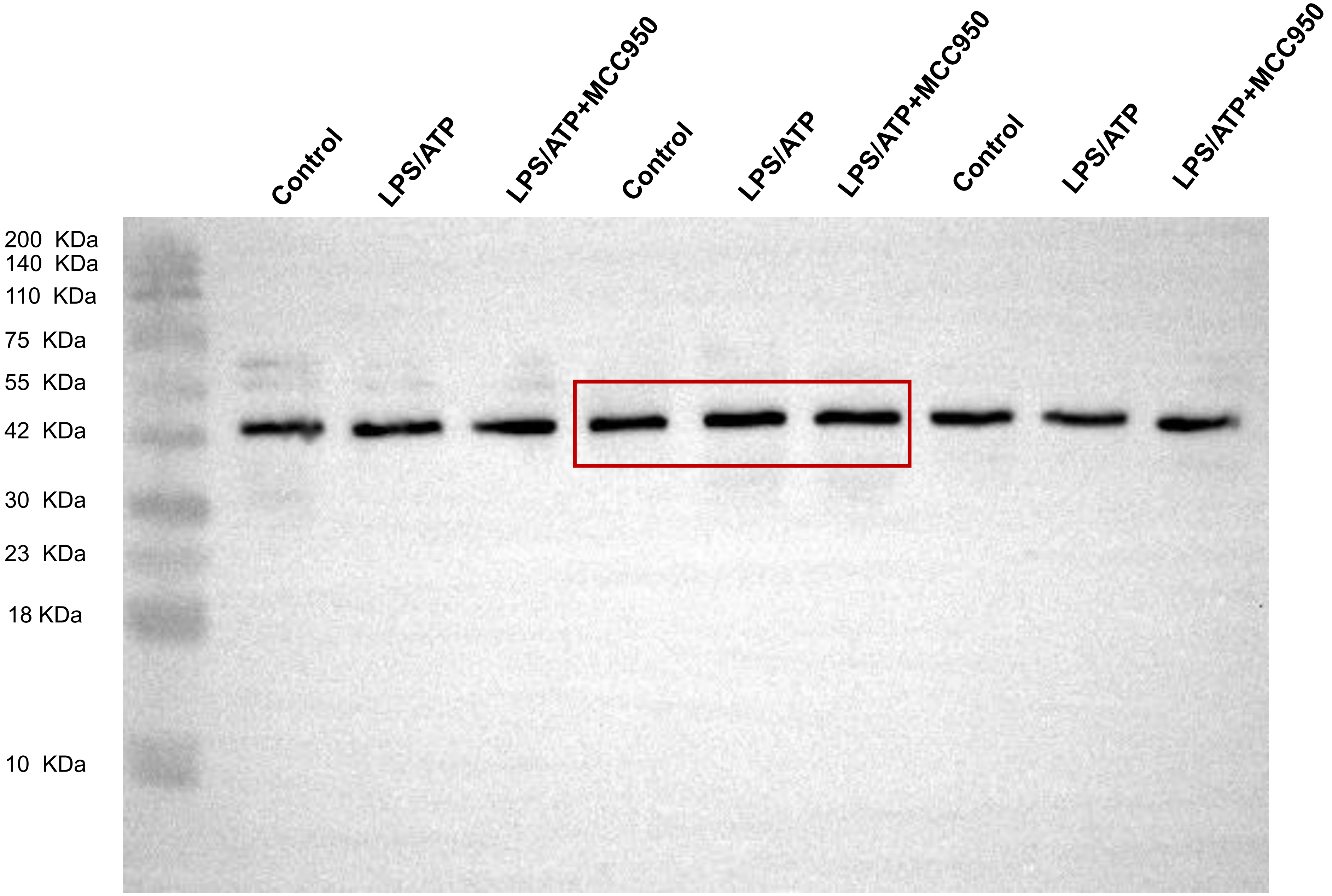

Figure 2B: Pro-caspase-1 (45 KDa)

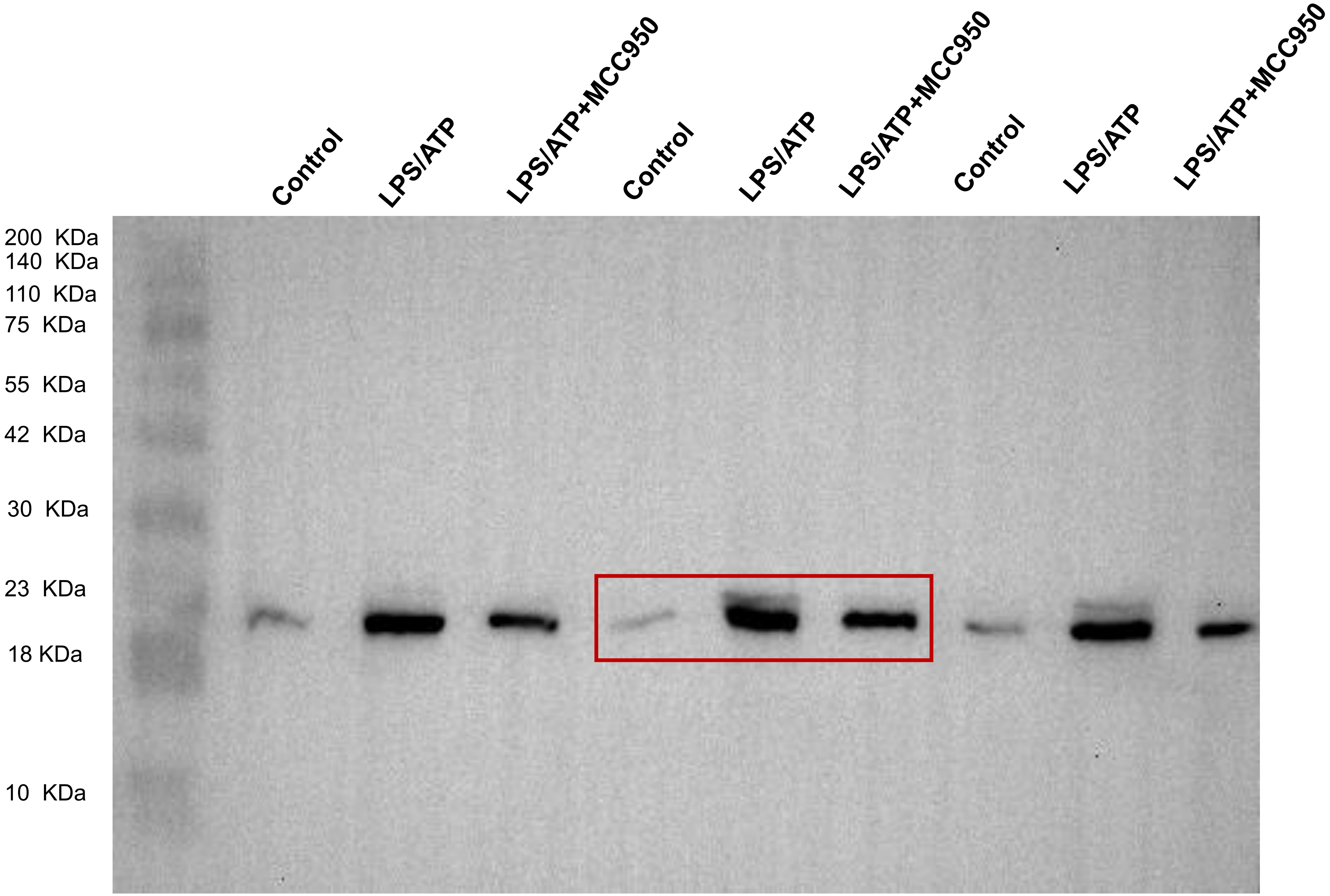

Figure 2B: Cleaved caspase-1 (20 KDa)

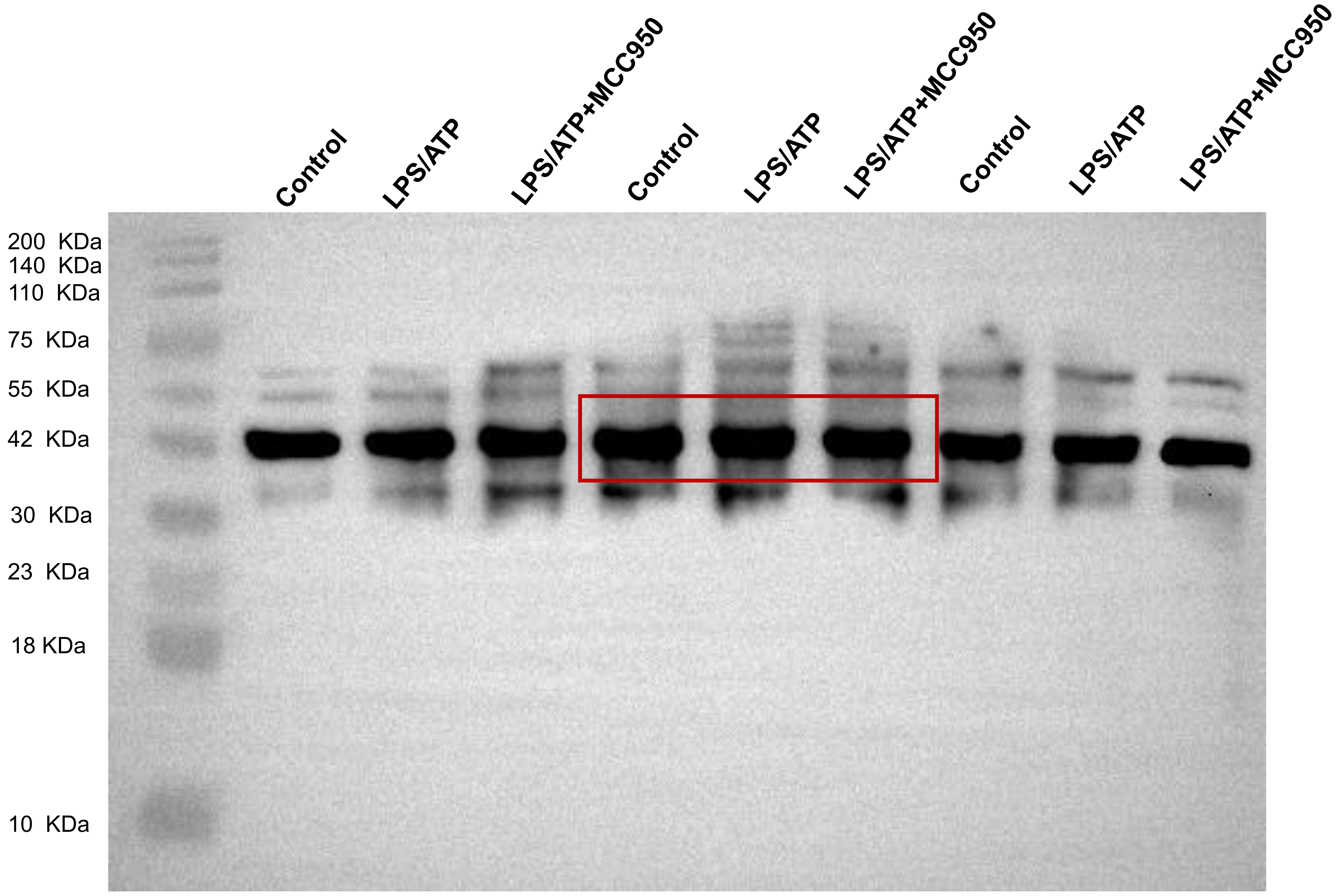

Figure 2B: β-actin (42 KDa)

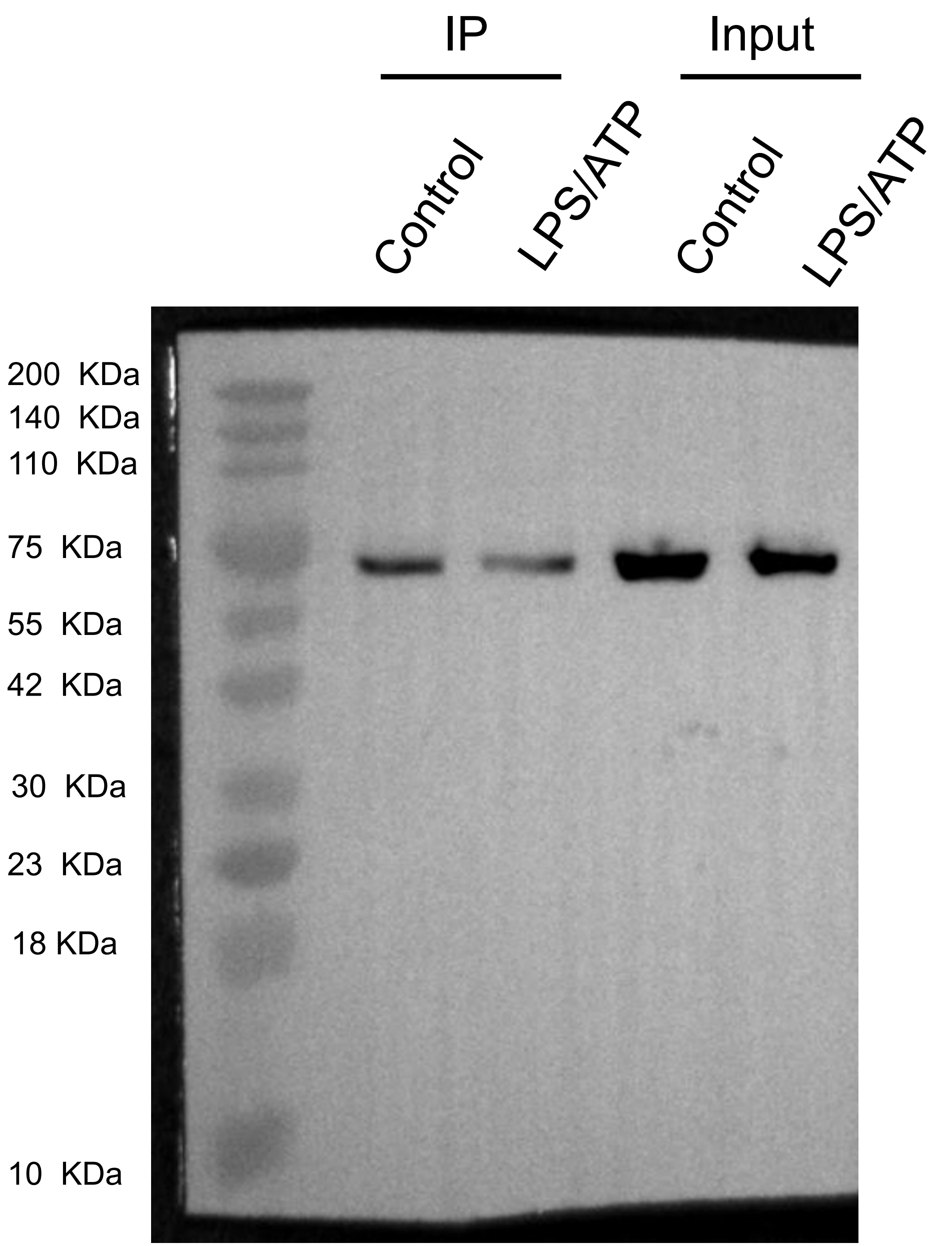

Figure 3B: FMR1 (72 KDa)

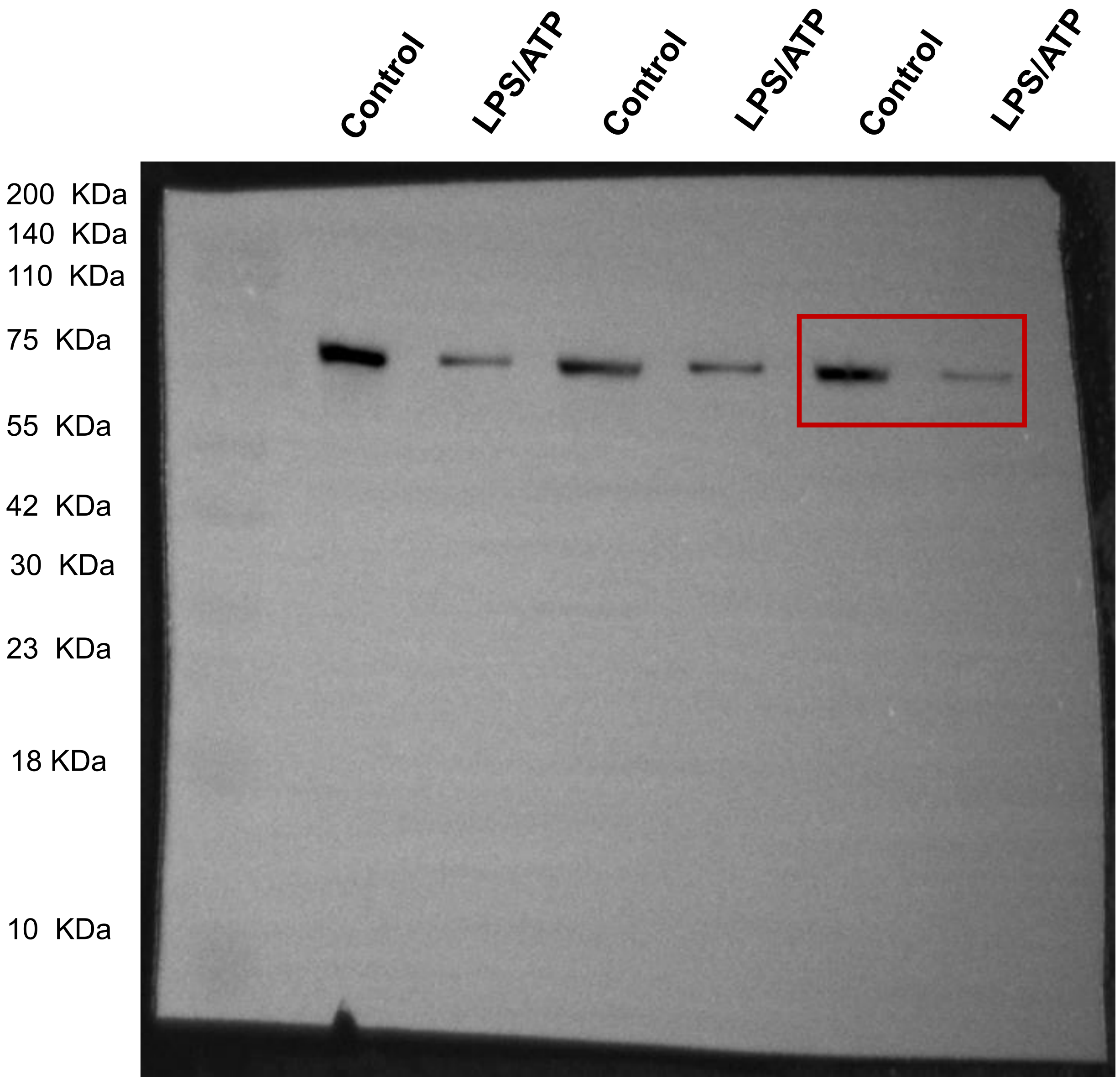

Figure 3D: FMR1 (72 KDa)

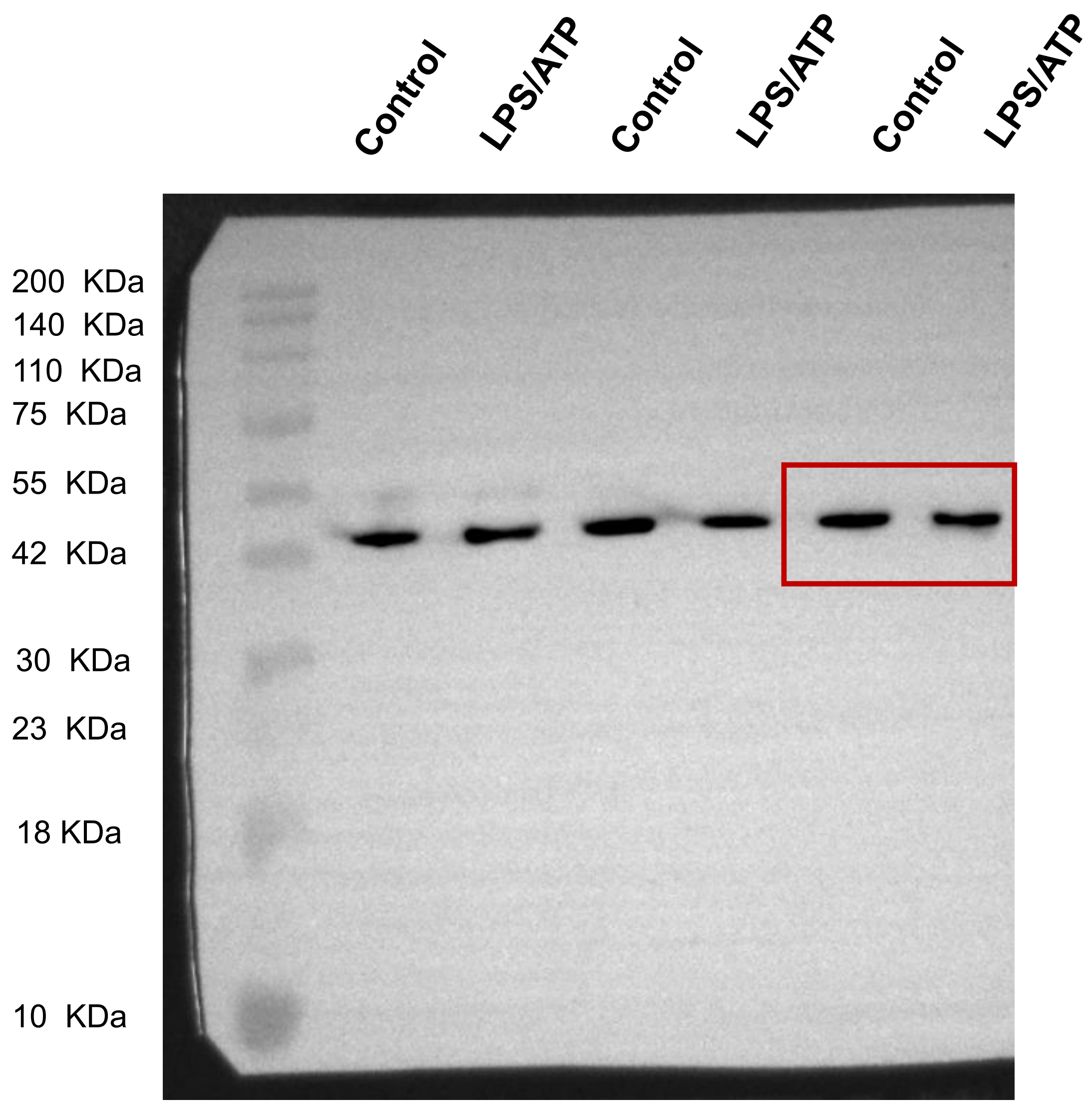

Figure 3D: β-actin (42 KDa)

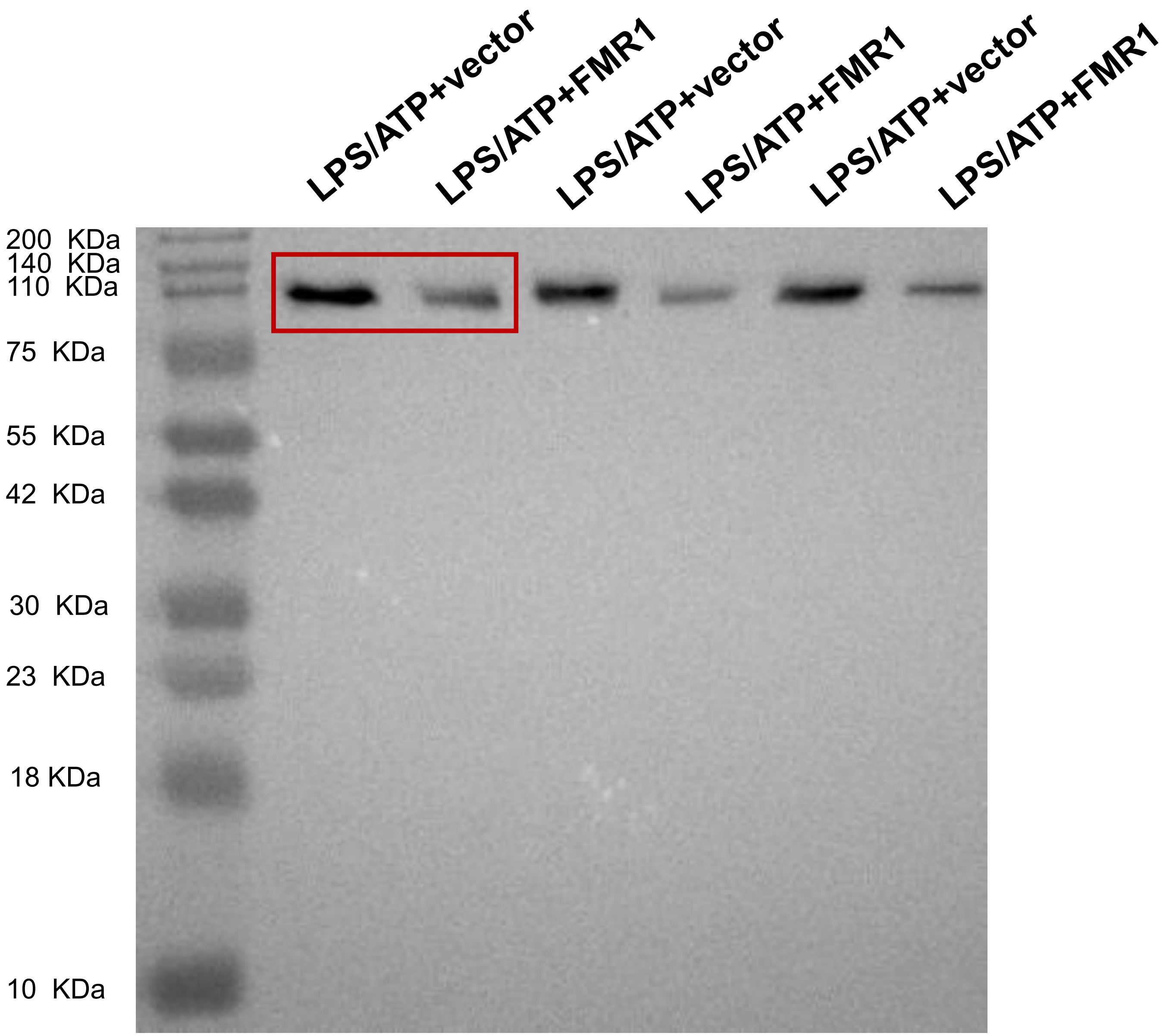

Figure 4C: NLRP3 (110 KDa)

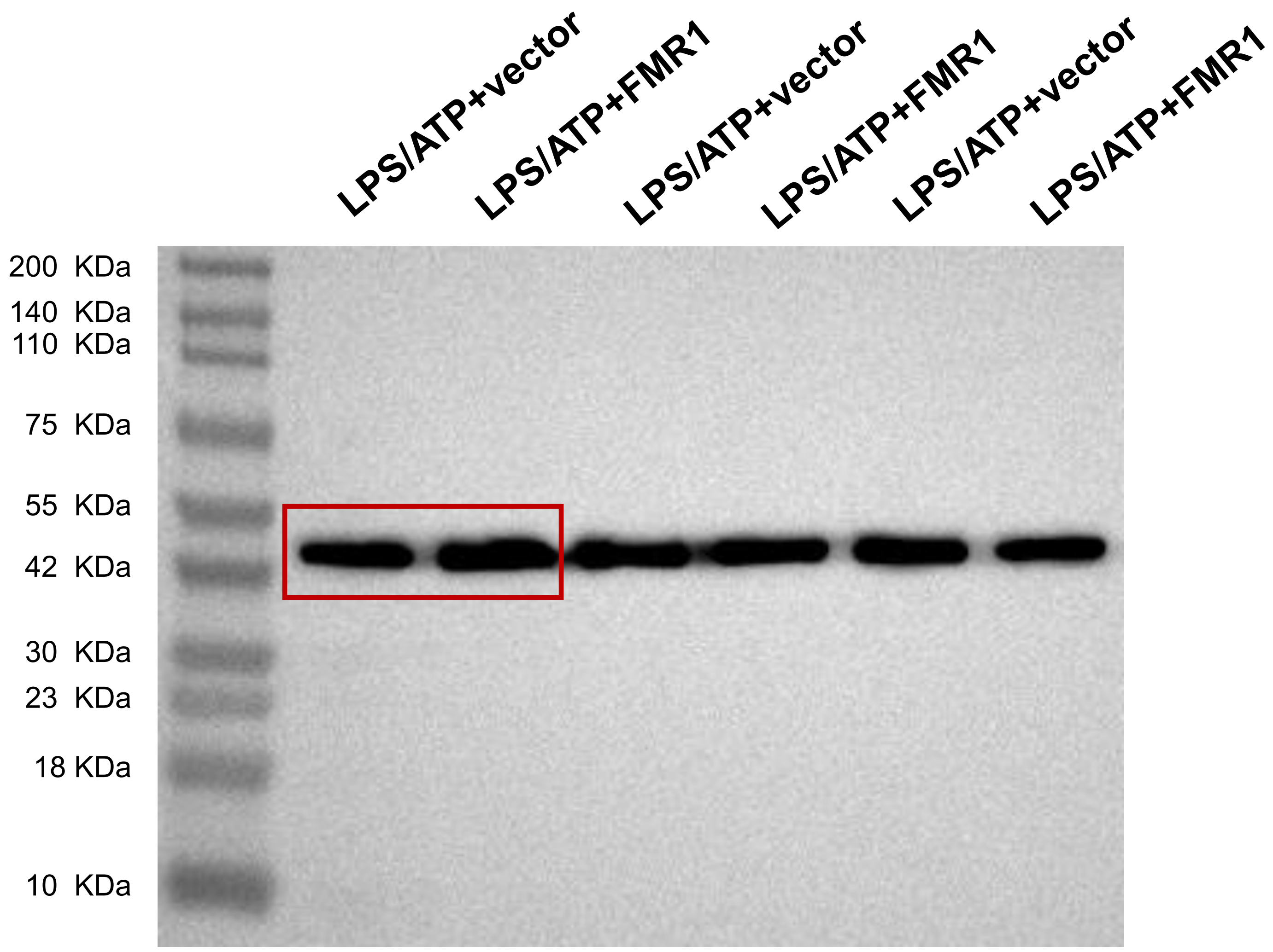

Figure 4C: β-actin (42 KDa)

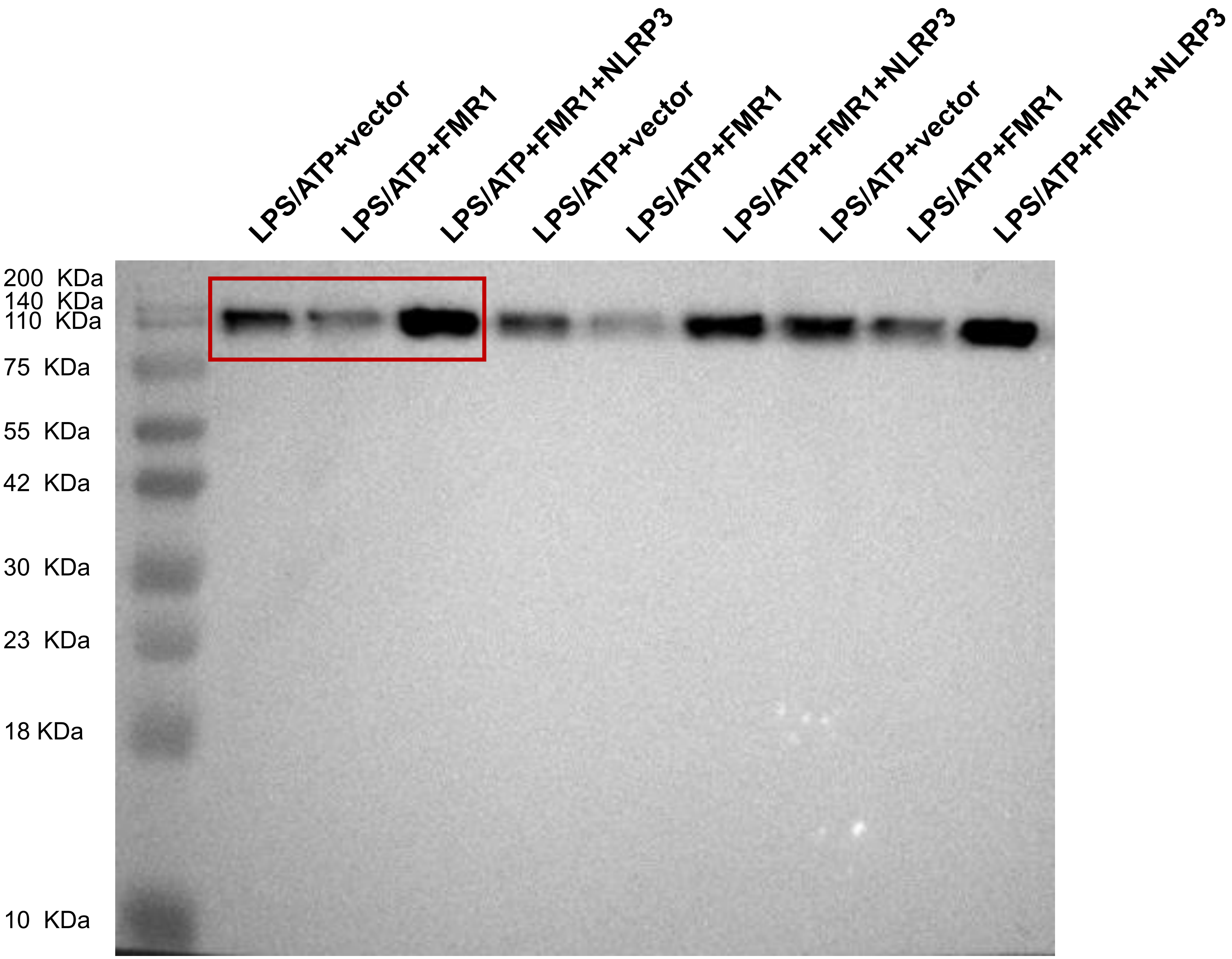

Figure 5A: NLRP3 (110 KDa)

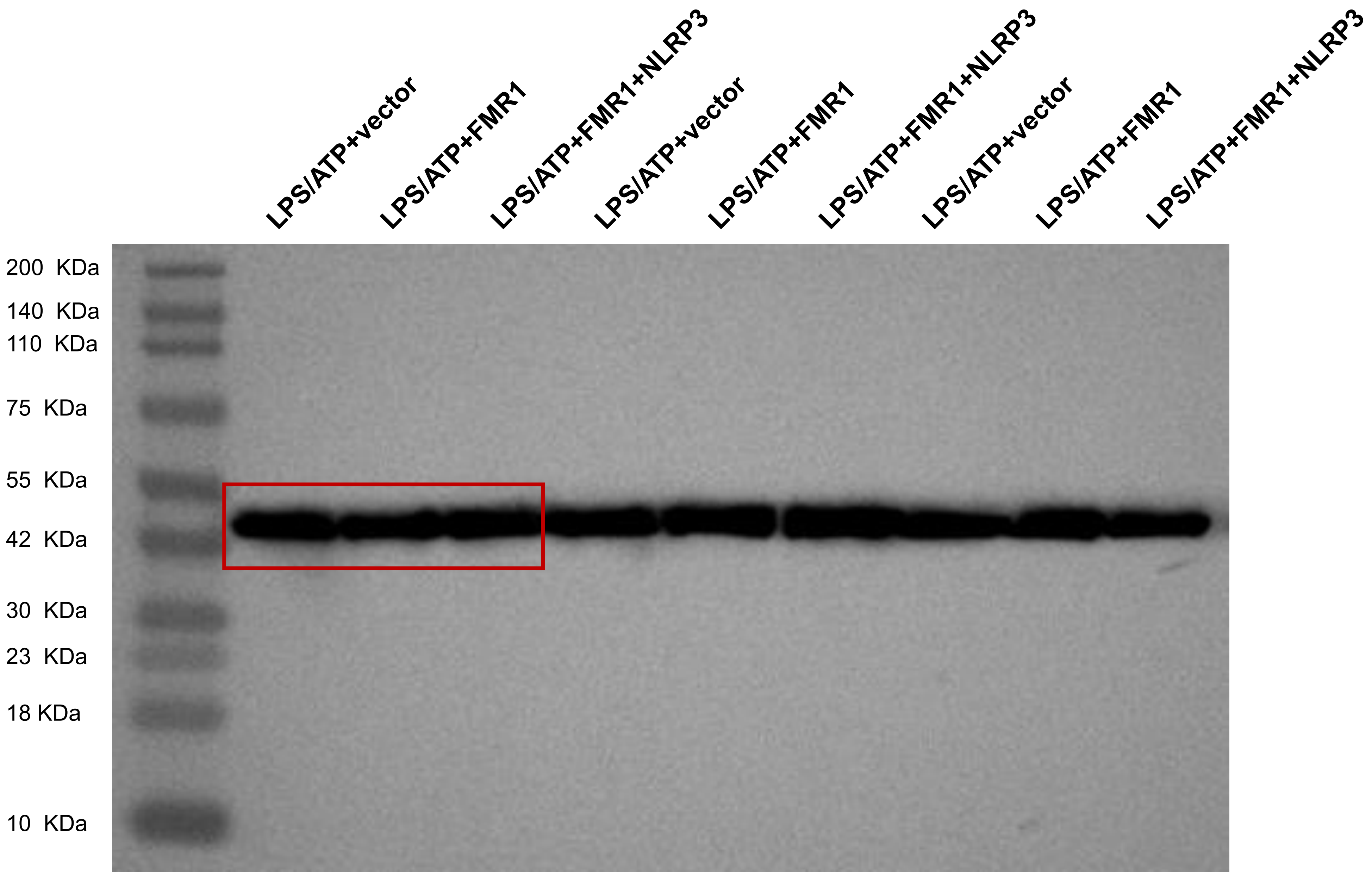

Figure 5A: β-actin (42 KDa)

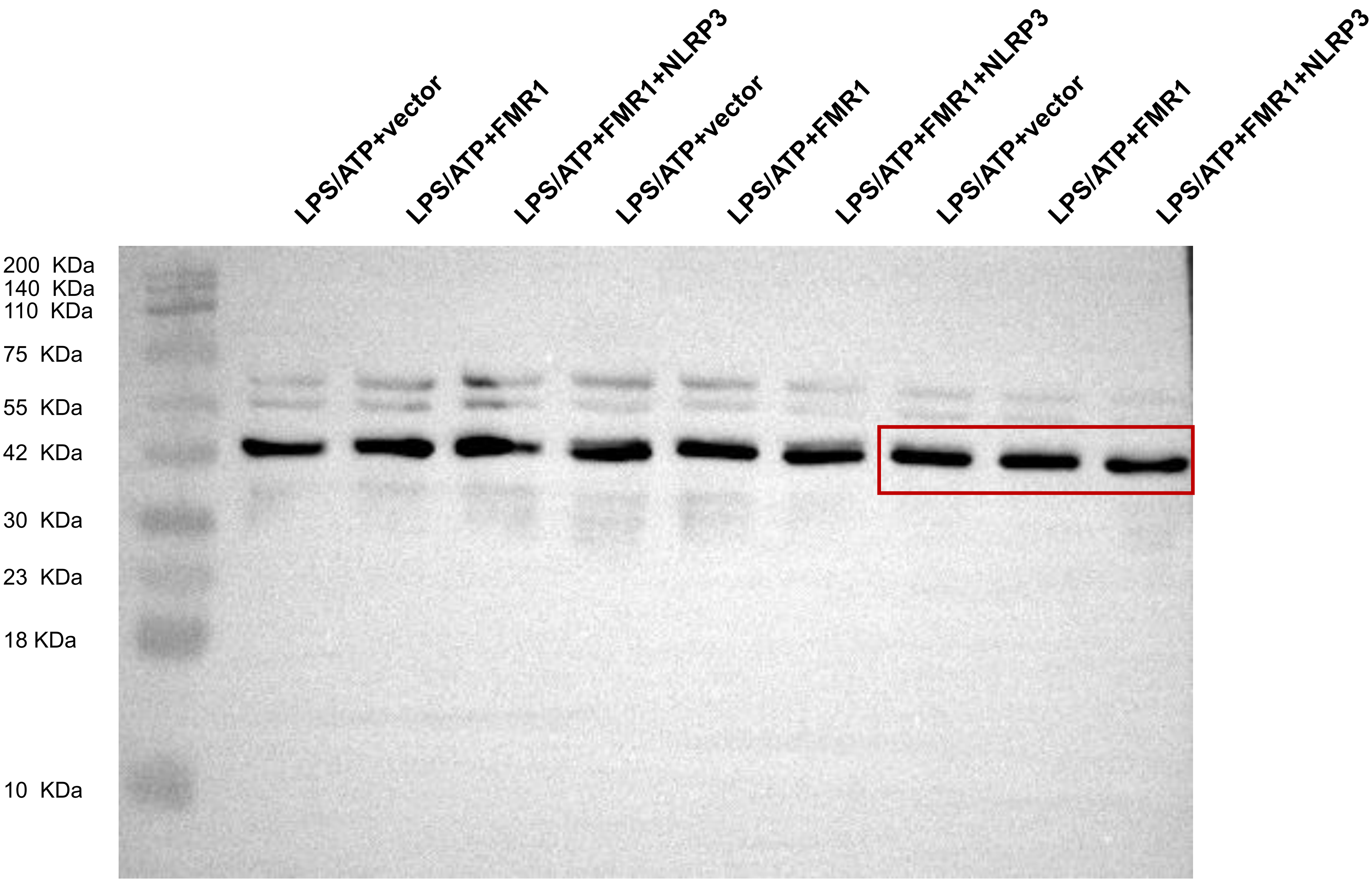

Figure 5A: Pro-caspase-1 (45 KDa)

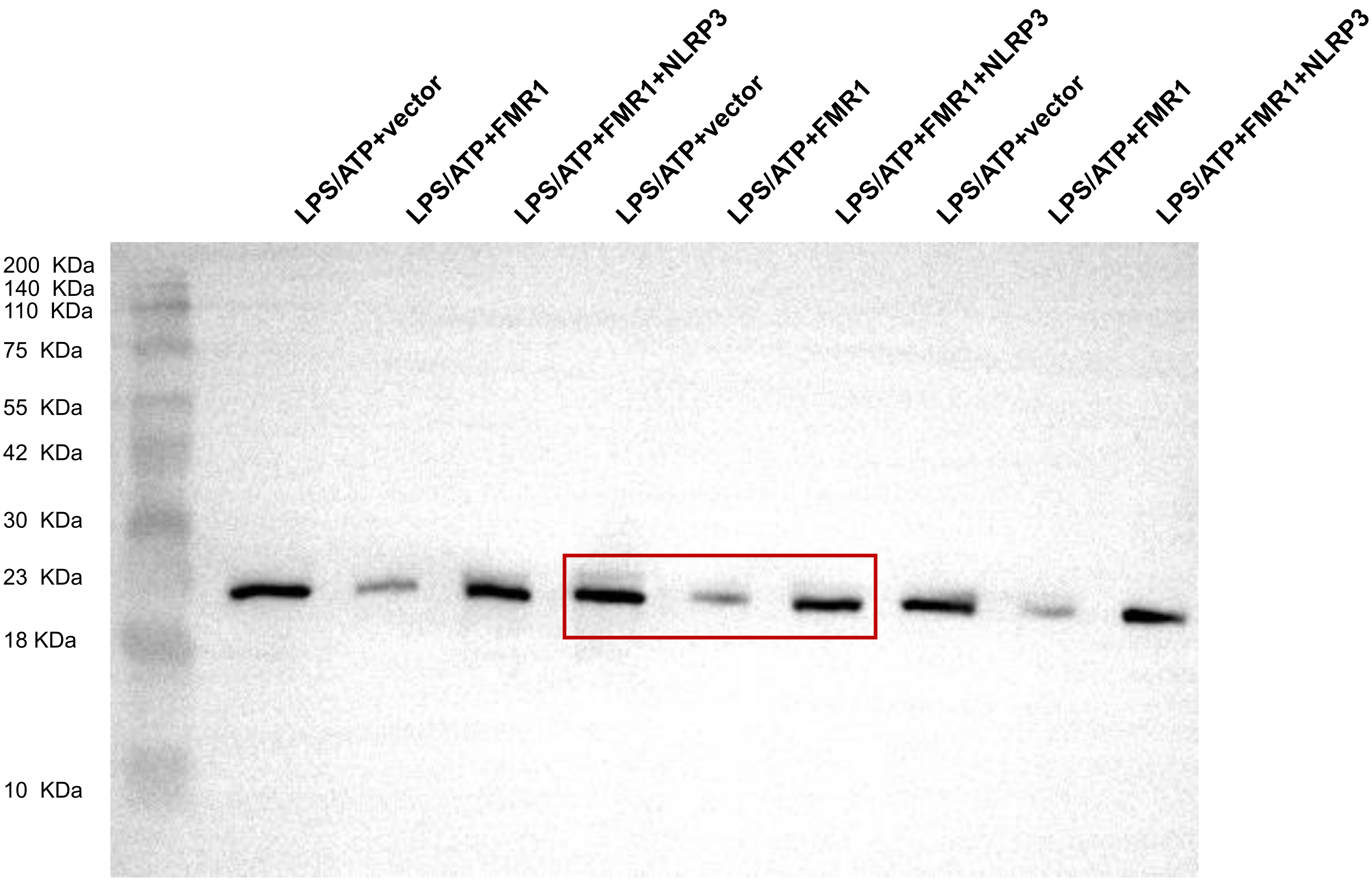

Figure 5A: Cleaved caspase-1 (20 KDa)

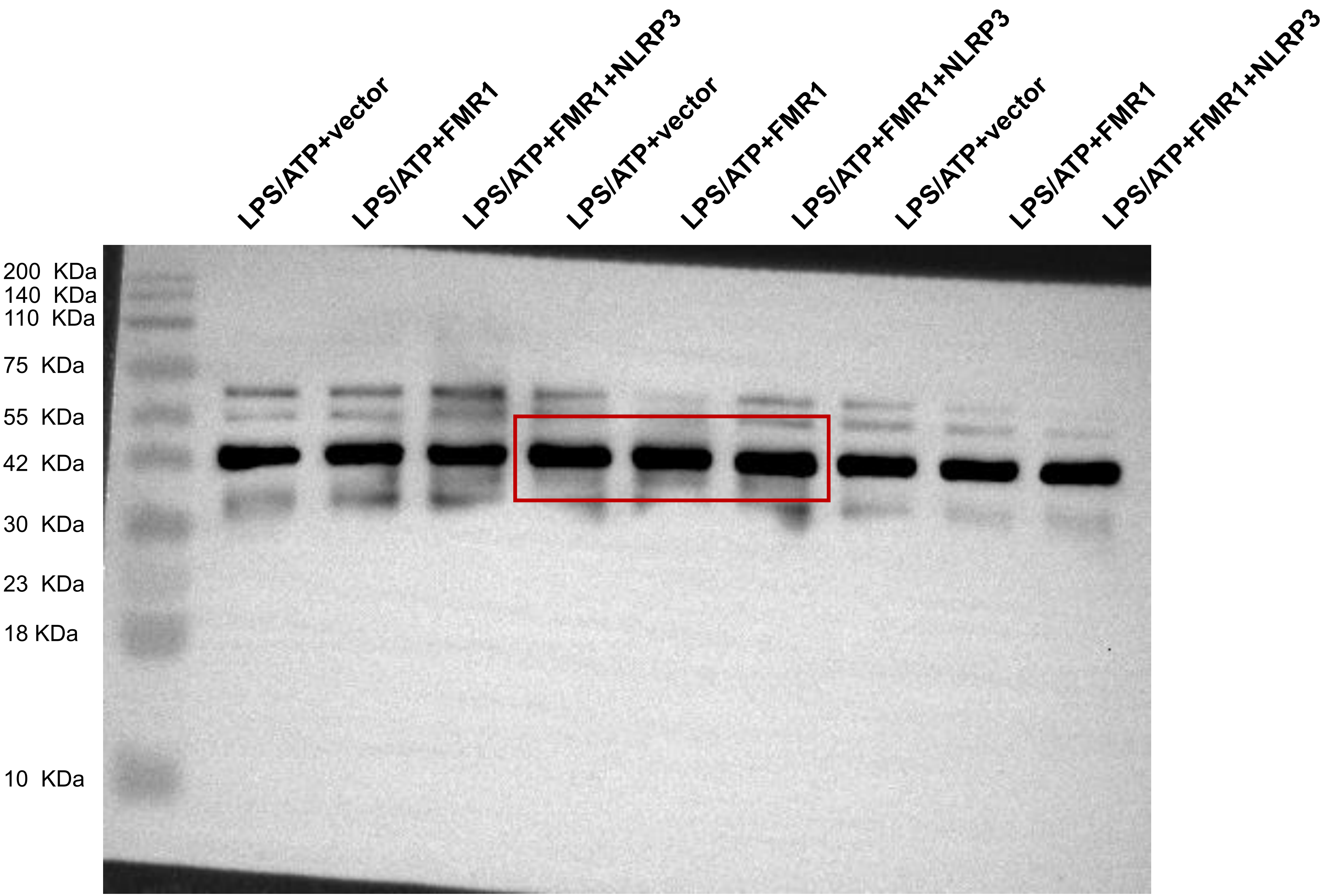

Figure 5A: β-actin (42 KDa)

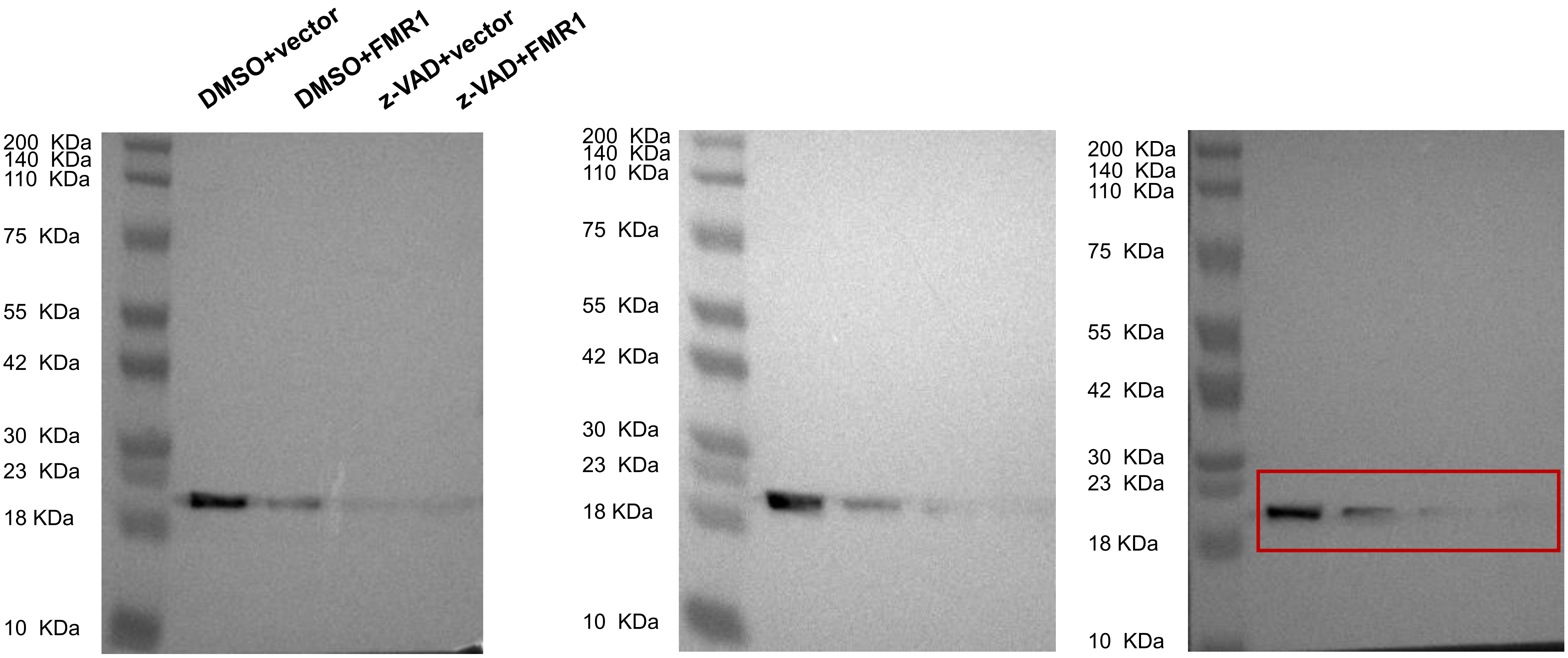

Figure S1: Cleaved caspase-1 (20 KDa)

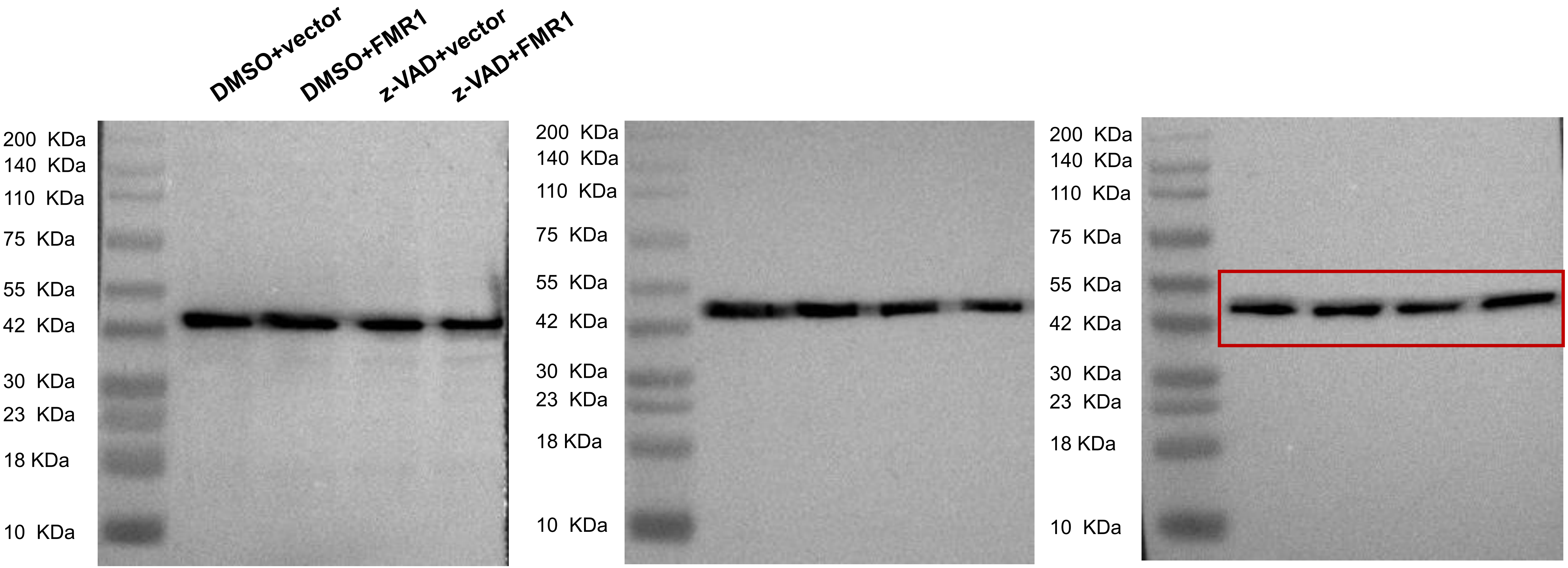

Figure S1: Pro-caspase-1 (45 KDa)

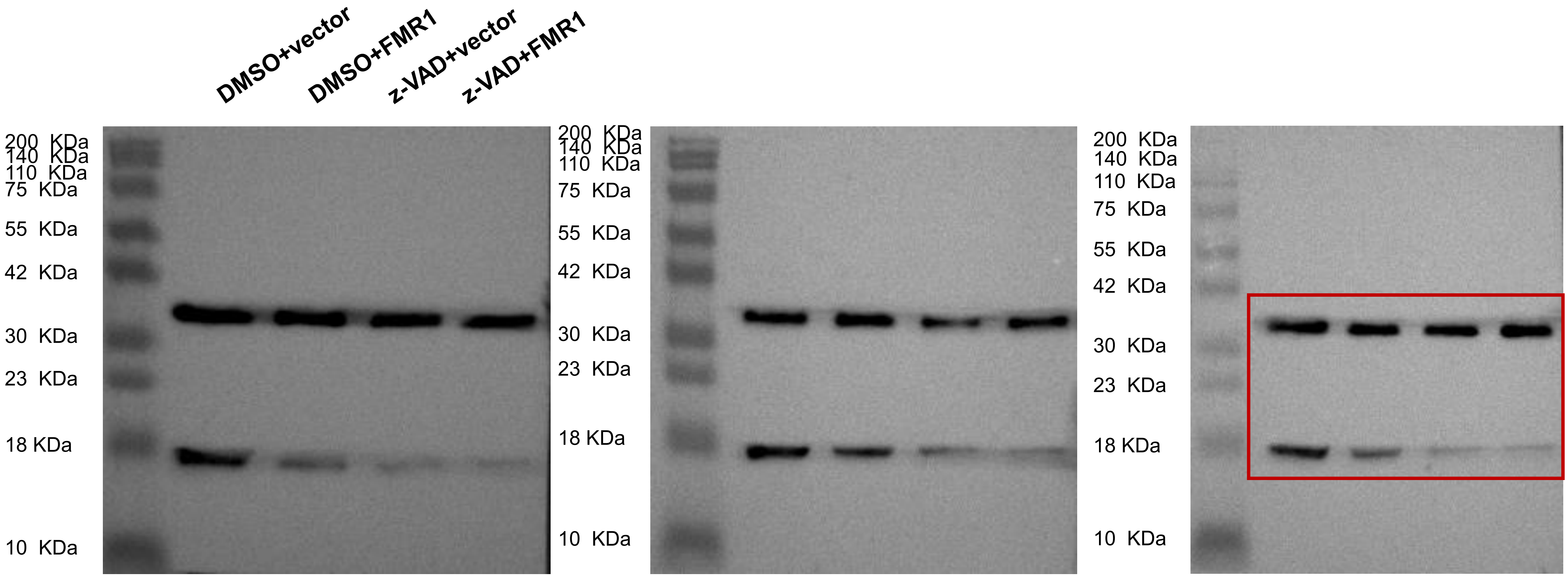

Figure S1: Pro-IL-1β\Mature IL-β (35/17 KDa)

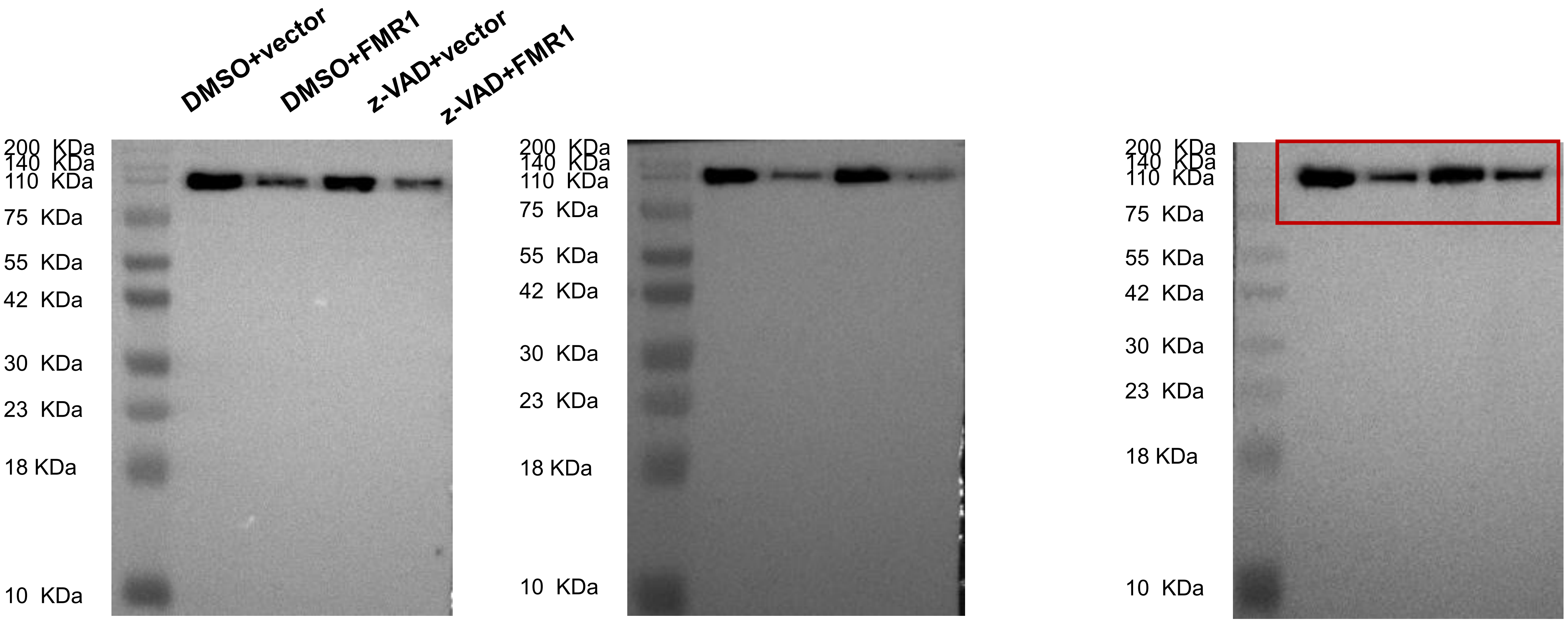

Figure S1: NLRP3 (110 KDa)

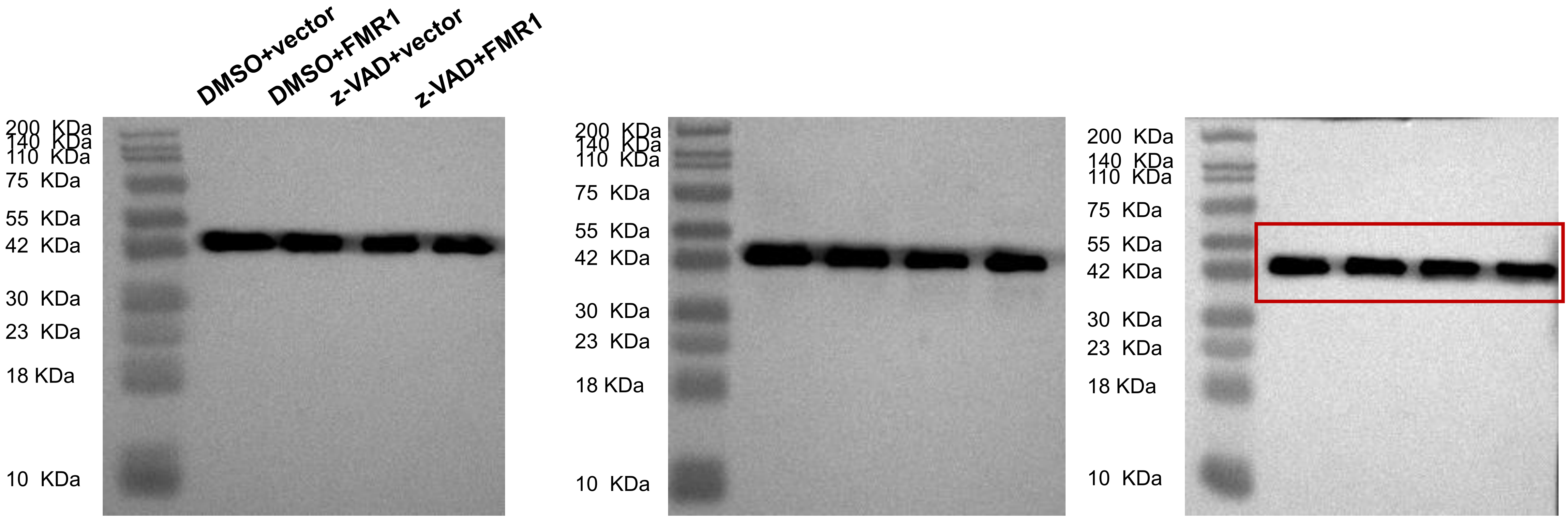

Figure S1: β-actin (42 KDa)

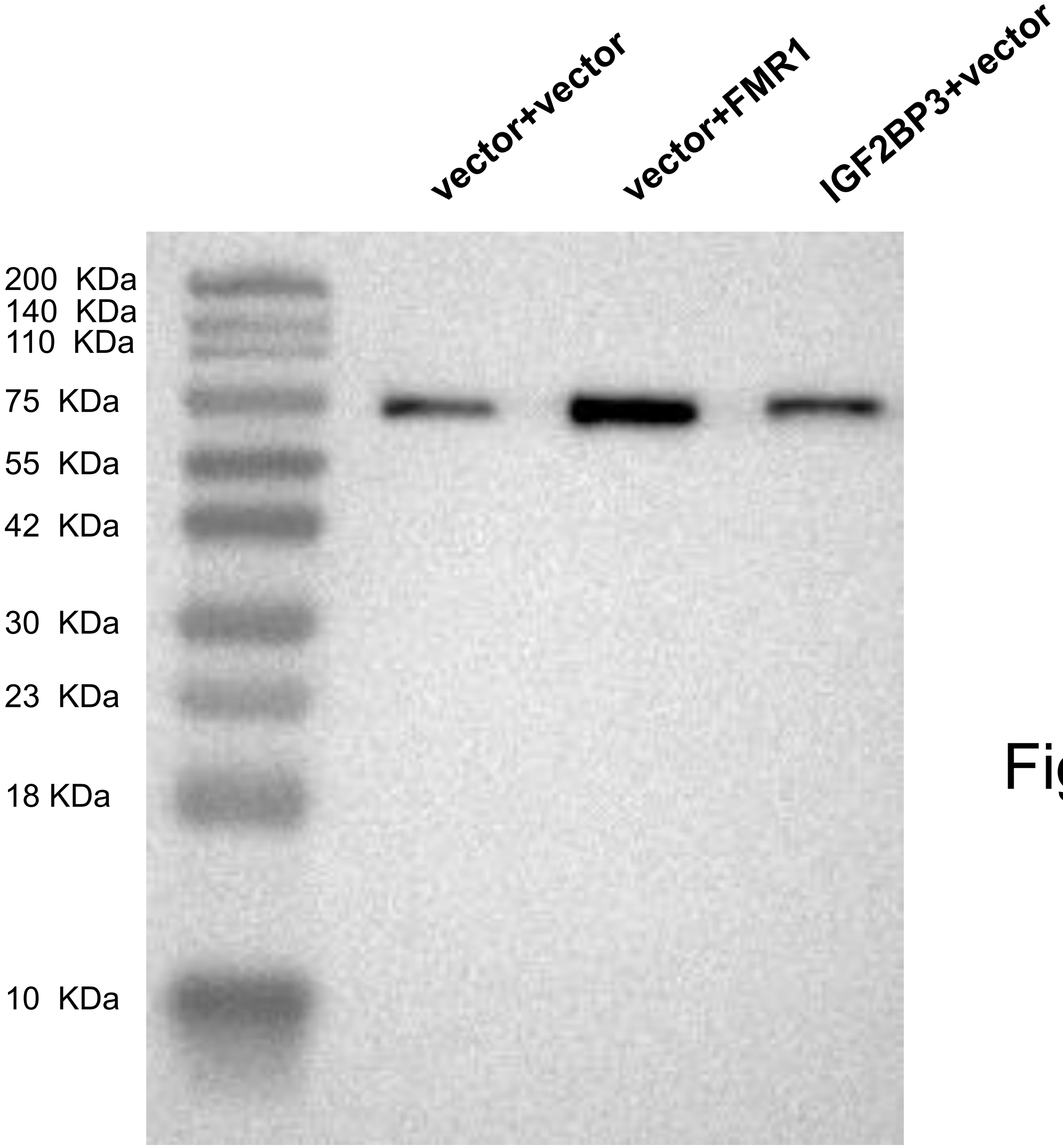

Figure S2: Input FMR1 (72 KDa)

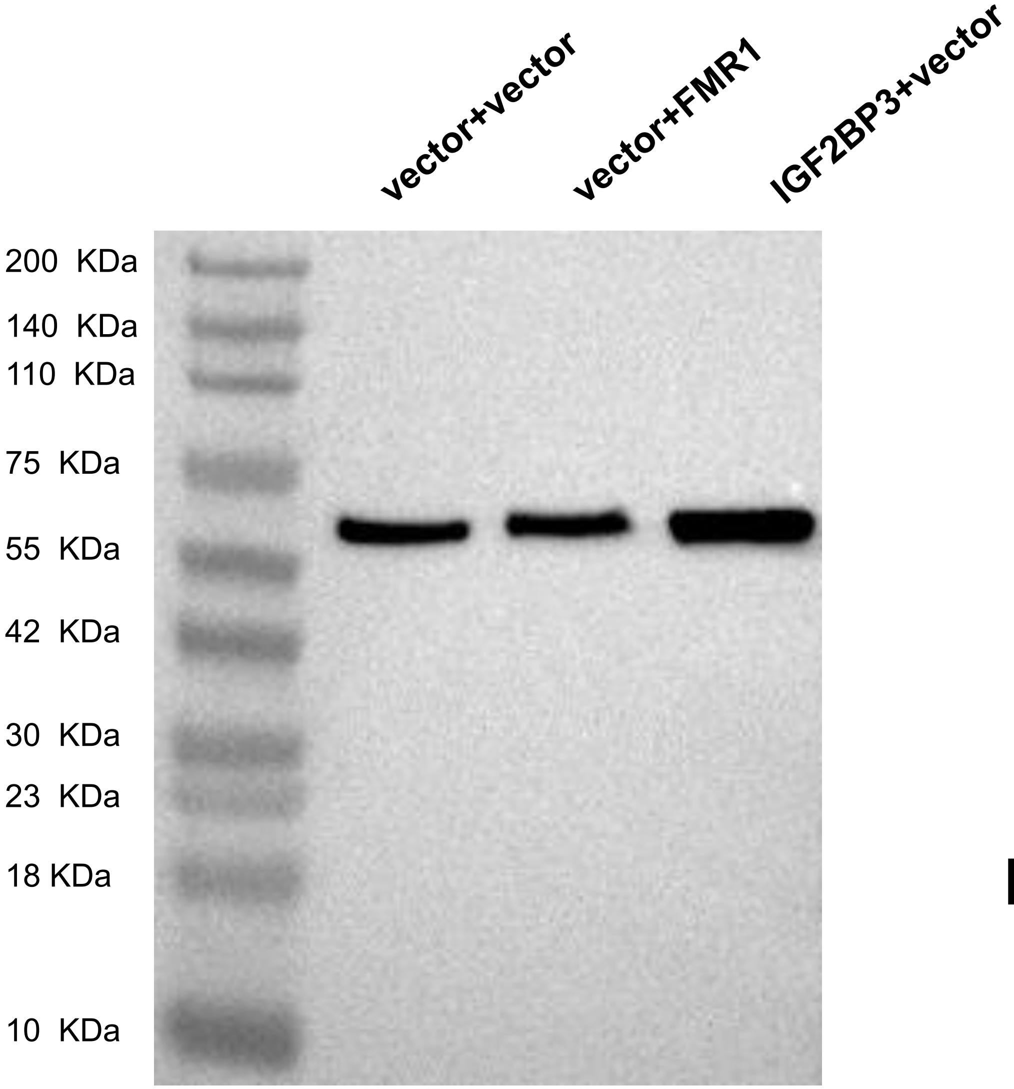

Figure S2: Input IGF2BP3 (64 KDa)

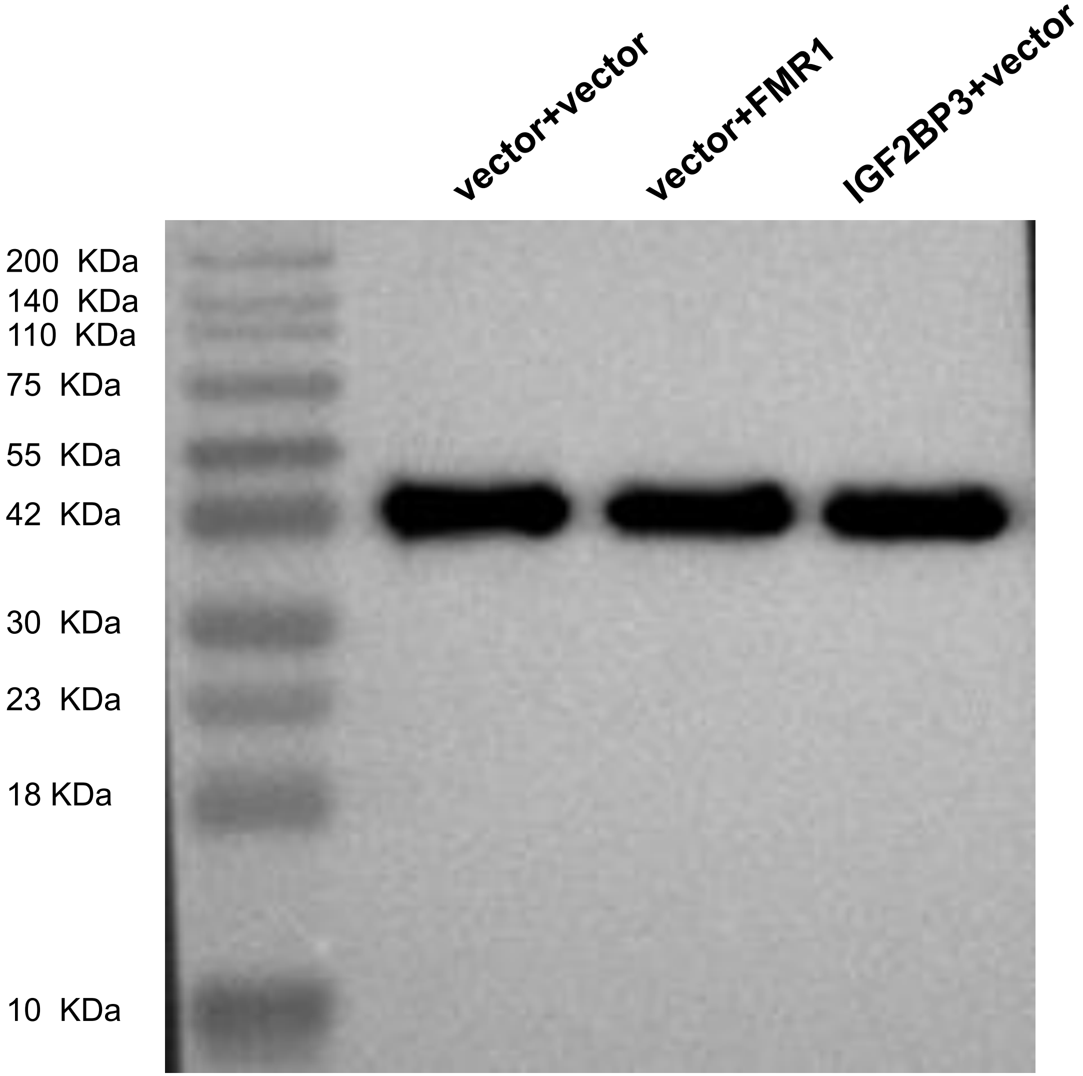

Figure S2: Input β-actin (42 KDa)

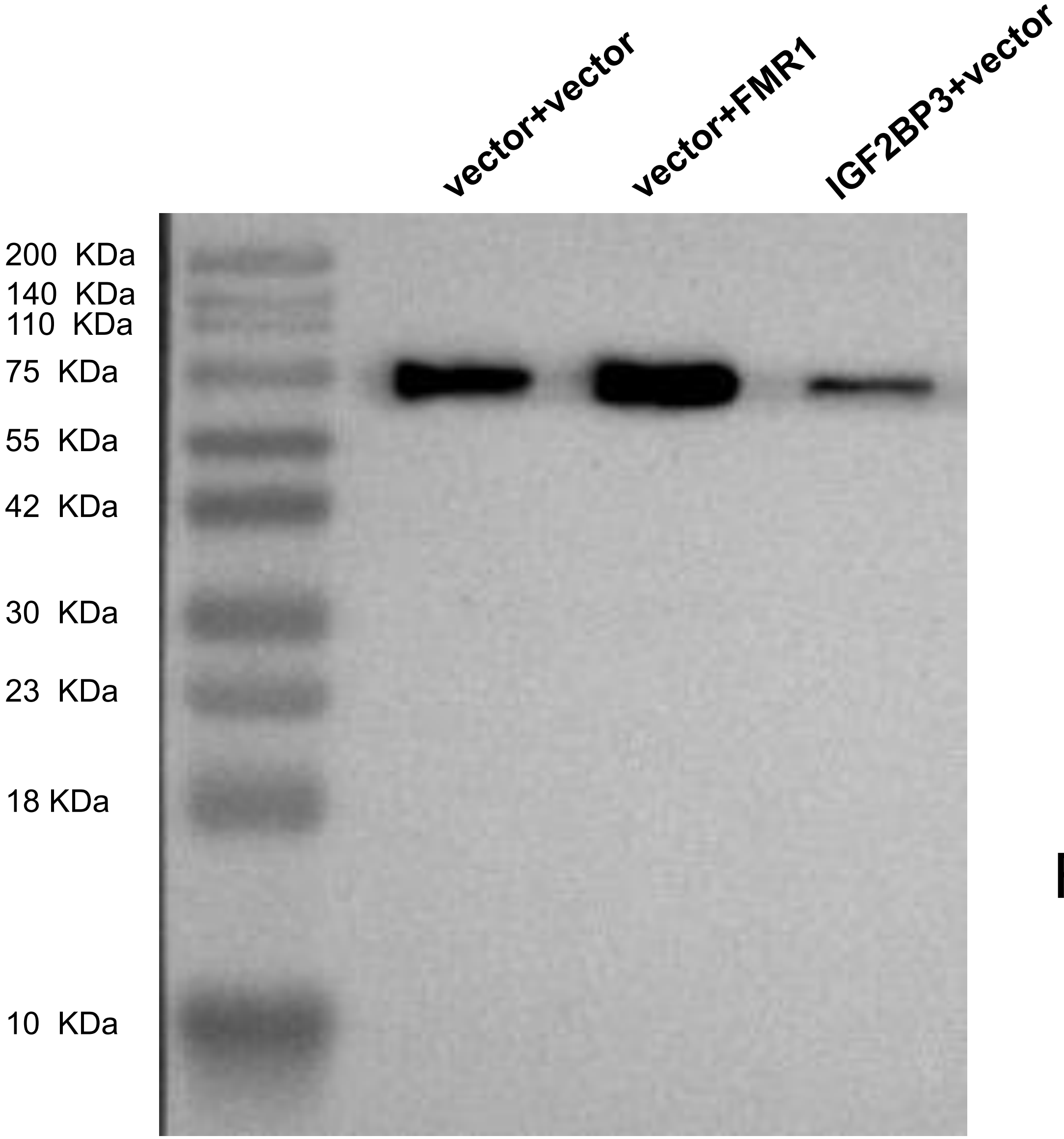

Figure S2: IP FMR1 (72 KDa)

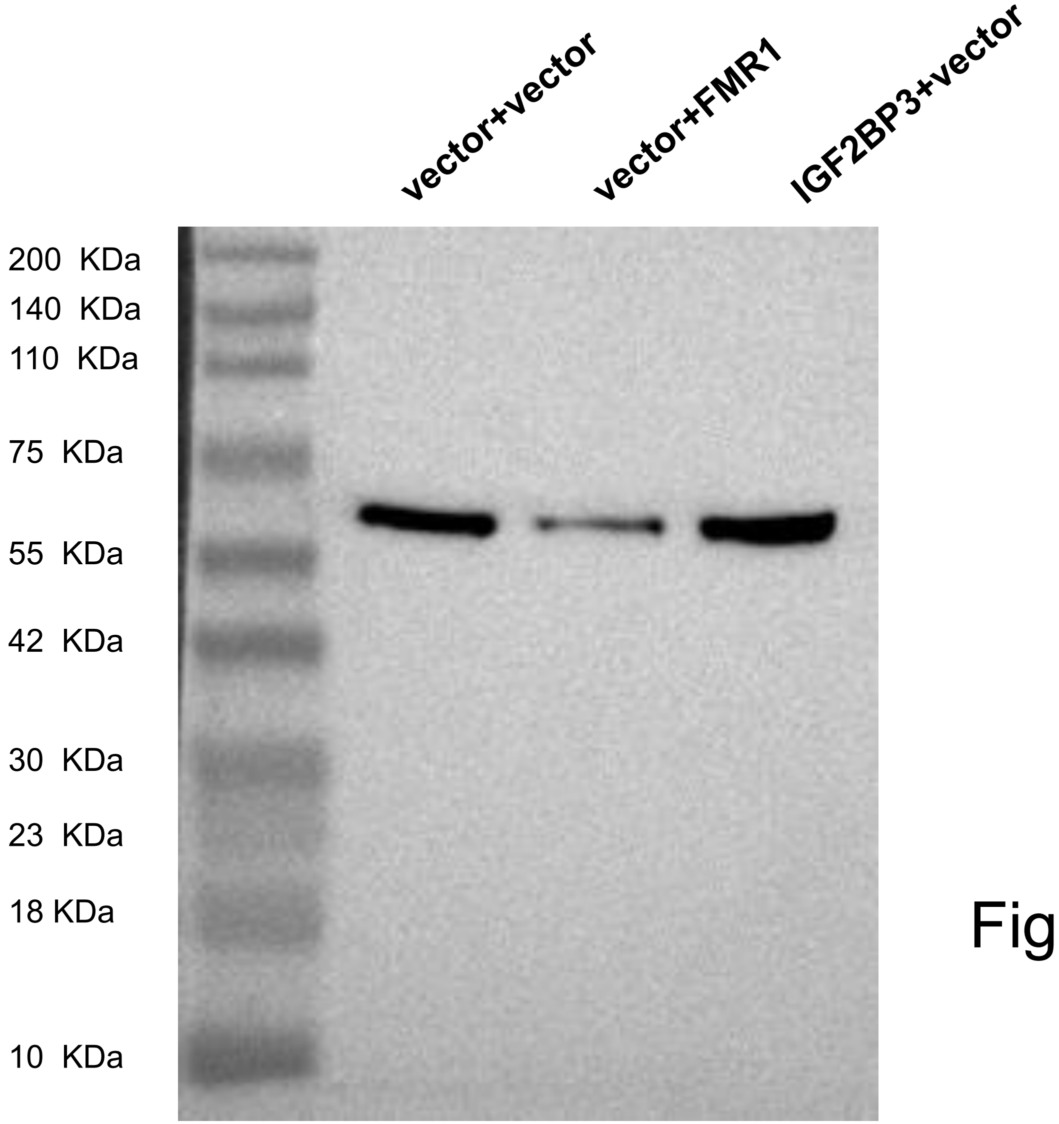

Figure S2: IP IGF2BP3 (64 KDa)
